# Multi-Target Pharmacological Effects of Asiatic Acid: Advances in Structural Modification and Novel Drug Delivery Systems

**DOI:** 10.3390/molecules30183688

**Published:** 2025-09-10

**Authors:** Xiaofan Dong, Tianyi Wang, Chenjia Gao, Yulong Cui, Lingjun Li

**Affiliations:** School of Pharmacy, Shandong University of Traditional Chinese Medicine, Jinan 250355, China

**Keywords:** asiatic acid, pharmacological effects, new dosage forms, structural modifications, medicinal plants

## Abstract

Asiatic acid is an ursane-type pentacyclic triterpenoid compound extracted from the Umbelliferae plant *Centella asiatica*. Studies have shown that asiatic acid exhibits a wide range of pharmacological activities, including anti-tumor, anti-inflammatory, hypoglycemic, antimicrobial, neuroprotective, and wound healing effects. Asiatic acid is currently used in clinical settings in the form of tablets, capsules, and ointments, primarily for treating inflammation as well as burns, keloids, and other skin disorders. However, its poor water solubility, rapid metabolism, and low oral bioavailability have limited its clinical application for other diseases. Therefore, improving its water solubility and bioavailability is a prerequisite for addressing the limitations of asiatic acid in clinical use. This review summarizes the pharmacological mechanisms of action of asiatic acid and explains the reasons for its limited clinical application. This review describes methods to improve bioavailability through structural modifications of asiatic acid and the development of new formulations. It also focuses on enhancing the pharmacological effects of asiatic acid through the development and utilization of novel formulations such as nanoformulations and hydrogel formulations, providing a theoretical basis for the clinical translation of asiatic acid and the further research and development of asiatic acid-based drugs.

## 1. Introduction

**Figure 1:**
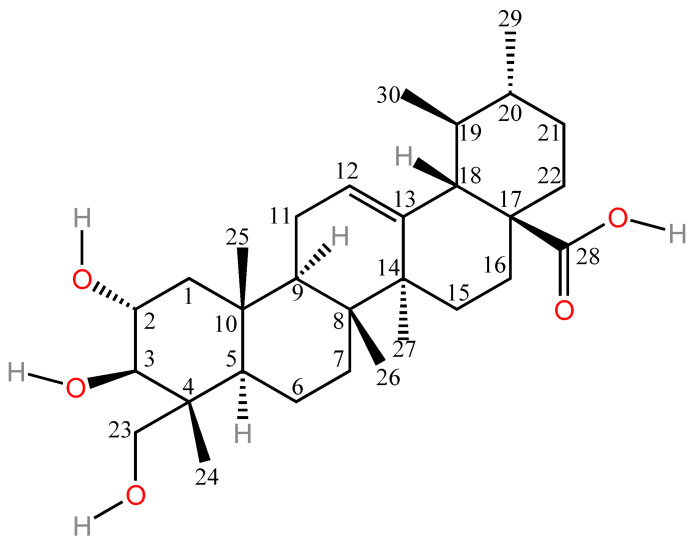
Chemical structure of asiatic acid.

**Figure 2:**
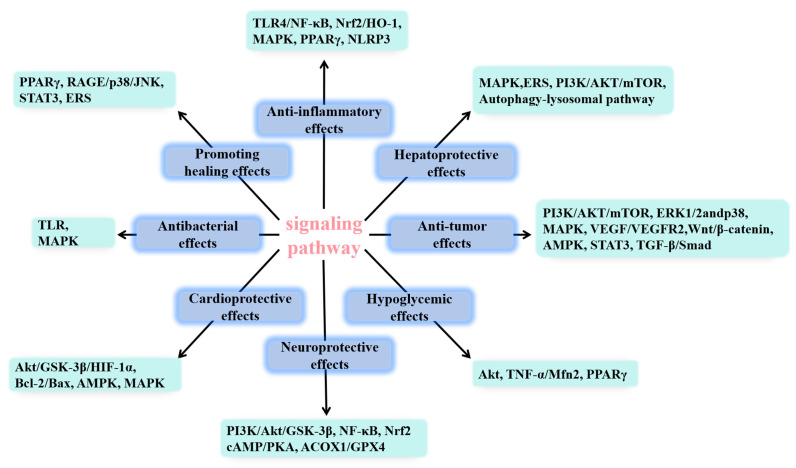
Specific Mechanisms of Action for AA Activation in Relation to Diverse Pharmacological Effects.

*Centella asiatica* (L.) *Urban* (*C. asiatica*) is a perennial herb in the Umbelliferae family [1], cold and bitter in property, with efficacy in clearing away heat and dampness detoxifying and eliminating swellings, mainly produced in Southeast Asia, Malaysia and other tropical swampy areas. Modern studies have shown that *C. asiatica* is rich in terpenoids, flavonoids and other chemical constituents, among which the triterpenoid asiatic acid (AA) is one of the main active components of *C. asiatica* (Figure 1), which exhibits various pharmacological effects including anti-inflammatory, hepatoprotective, anti-tumor, hypoglycemic, neuroprotective, and anti-bacterial activities, as well as wound healing promotion. The targets of AA activation vary depending on the specific pharmacological effects being exerted, as illustrated in Figure 2. Therefore, biological function analysis of AA can better elucidate its significance in the therapeutic field and provide a better reference for the clinical use of AA and the development of new drugs. The nonpolar part of its structure leads to poor water solubility of AA (10 µg·mL^−1^), which in turn leads to its low oral utilization (C_max_ = 0.098 µg·mL^−1^, AUC_0–12h_ = 0.61 ± 0.25 µg·h·mL^−1^), limiting its clinical application [2]. Therefore, it is essential to improve the biological activity of AA through structural modification. For instance, the introduction of amide groups at the C-2, C-3, and C-23 positions of AA for hydroxyacetylation [3,4,5], the esterification of amino acids at the C-28 position (the indication of C atoms can be found in Figure 1), and the establishment of novel intermolecular hydrogen bonds can significantly enhance the antitumor activity, cytotoxicity, and selectivity of AA [6,7]. This is because intramolecular hydrogen bonds can modify the lipid solubility, membrane permeability, and target binding efficiency of AA; hydroxyacetylation can similarly enhance the lipid solubility of AA to facilitate transmembrane transport; and esterification of amino acids at the C-28 position can improve both water solubility and achieve slow release of the active molecule via enzymatic digestion. Collectively, these modifications contribute to enhanced bioavailability. In addition, encapsulating AA within nanoparticles [8], liposomes [9], or other polymeric materials and integrating them into novel formulations can enhance its physical properties. These improvements include increasing the solubility of AA, enhancing its skin permeability [10], prolonging its retention time in the intestines following systemic administration [11], and extending the drug release duration [12]. Consequently, these modifications collectively contribute to improved bioavailability. The structural modification of AA, as well as the utilization of novel delivery systems to enhance its pharmacological activity, is both primarily focused on addressing the issue of AA’s low aqueous solubility, thereby improving its bioavailability. In this paper, we summarize the AA-related literature in recent years and systematically review the pharmacological activities and new dosage forms of AA and its derivatives to help researchers understand the current status of AA more comprehensively and to provide a theoretical basis for further research on AA.

## 2. Pharmacological Effects of Asiatic Acid

### 2.1. Anti-Inflammatory Effects

Inflammatory response is a central regulatory hub in the pathological processes of many diseases [13]. AA has significant anti-inflammatory properties, and its anti-inflammatory effects are exerted through multi-target mechanisms, including regulating the level of inflammatory factors, activating signaling pathways, interfering with enzyme synthesis and expression, or regulating the content of mitochondrial reactive oxygen species (ROS), to treat endometritis, neuroinflammation, rheumatoid arthritis, and many other inflammatory diseases. Nuclear factor erythroid 2-related factor 2 (Nrf2) is one of the important systems in response to oxidative stress and inflammation [14]. The mechanism by which AA alleviates inflammation by modulating the Nrf2 signaling pathway is of great interest. Risya Cilmiaty et al. [15] showed that AA was able to activate the Nrf2 signaling pathway in a dose-dependent manner and reduce the levels of inflammatory factors, such as IL-17, Th1 and Th17, thereby inhibiting oxidative stress for anti-inflammatory effects by studying a lipopolysaccharide (LPS)-induced model of endodontitis in rats. In a recent study, Zhong [16] found that AA was able to effectively alleviate *Salmonella*-induced colitis by enhancing the function of the intestinal mucosal barrier and decreasing the expression of inflammatory factors. AA intervention reduced inflammatory cell infiltration and significantly increased claudin-2 and claudin-7 expression, protecting the intestinal mucosa of mice with *Salmonella*-induced enteritis. In addition, AA intervention significantly reduced the expression levels of IL-1β, IL-6, and TNF-α mRNA and inhibited the inflammatory response. AA demonstrates great potential as an adjunctive drug for salmonellosis control.

A large number of studies have reported that AA can improve inflammation through multiple targets and pathways. Extensive research supports the core anti-inflammatory mechanism, which may lie in the activation or inhibition of upstream mechanisms, thereby regulating the expression of downstream factors and ultimately achieving a certain anti-inflammatory effect. In summary, AA primarily reduces the expression of downstream pro-inflammatory factors (TNF-α/IL-1β/IL-6) and inflammatory mediators (COX-2/iNOS) through mechanisms such as activating upstream antioxidants (Nrf2), inhibiting pro-inflammatory factors (NF-κB/NLRP3), and regulating metabolic pathways (PPARγ). The key anti-inflammatory mechanisms are summarized in Table 1.

Studies on various types of inflammation have found that AA inhibits the release of inflammatory factors through multiple targets and pathways, with regulation of the NF-κB pathway being the most common pathway. At the same time, the anti-inflammatory effects of AA mostly stem from short-term cellular effects (such as apoptosis and proliferation) and the regulation of signaling pathways. Some studies have not involved animal experiments or the long-term efficacy of AA in chronic inflammation. Future research should explore the long-term efficacy of AA in chronic inflammation animal models and assess its impact on chronic inflammation biomarkers. Additionally, the potential for combining AA with other anti-inflammatory drugs should be explored.

### 2.2. Hepatoprotective Effects

The liver is responsible for a variety of physiological activities in the body, including detoxification, antioxidant activity and immunomodulation [30]. AA has now been reported to be a hepatoprotective agent with significant effects on liver protection. Zhu et al. [31] demonstrated experimentally that AA was able to ameliorate hepatic histopathological changes, reduce aminotransferase activity, inflammation, oxidative stress injury, and the levels of ALT and AST, important serum markers of hepatic injury, and effectively reverse rifampicin (RFP)-and isoniazid (INH)-induced liver injury. Subsequently, comprehensive transcriptomic and metabolomic analyses revealed that AA was able to down-regulate the expression level of *Degs1* to affect the levels of key metabolites (dihydroceramide, sphingomyelin, and galactosylceramide), as well as inhibit the phosphorylation of ERK, JNK, and p38 MAPK to block inflammatory signaling, and synergistically attenuate RFP/INH-induced hepatic injury through the regulation of sphingolipid metabolic pathway and inhibition of MAPK signaling pathway, confirming the hepatoprotective effect of AA. However, this mechanism has mainly been validated in acute drug-induced liver injury models, and its efficacy in chronic liver disease or liver injury caused by other etiologies remains to be explored. Pang et al. [32] showed that acute liver injury (ALI) model mice significantly improved the expression of proteins such as p-PERK, p-eIF2α, ATF4, and p-JNK, reduced endoplasmic reticulum stress (ERS), and promoted the maturation of autophagic lysosomes in LO2 cells to promote hepatocyte autophagy after intervention with AA to alleviate ALI. Wei et al. [33] found that AA was able to reduce CCl_4_-induced hepatic fibrosis in a dependent manner, and the mechanism may be related to the inhibition of the PI3K/AKT/mTOR signaling pathway, the reduction of cellular oxidative stress, and the amelioration of inflammation. In another study [34], AA was shown to inhibit hepatic fibrosis by decreasing levels of collagen type 1 and pro-collagen type 1. Recent studies have found that AA promotes the degradation of HBx proteins through the autophagy-lysosome pathway, inhibits the binding of HBx to hepatitis B virus (HBV), and reduces the transcriptional activity of cccDNA, exerting an anti-HBV effect [35], which may provide a breakthrough point for curing hepatitis B in the future. However, this research is still in the basic research stage, and its antiviral efficacy compared to other existing therapies needs to be verified through other models and clinical trials. In general, AA reduces liver damage through multiple targets, but these findings are based on animal models and cell experiments, lacking clinical trial support. The translational pathway from basic experiments to clinical application remains unclear. Future efforts should focus on addressing these gaps and validating the clinical value of AA in treating liver damage.

### 2.3. Anti-Tumor Effects

AA has remarkable anti-tumor activity, inhibiting a variety of cancers such as breast cancer, nasopharyngeal carcinoma, lung cancer, etc., through a multi-pathway and multi-target approach. Tian et al. [36] showed that AA (50 mg·kg^−1^) was able to reduce tumor volume and the number of lung nodules in breast cancer model mice. Another study showed [37] that AA (50 mg·kg^−1^) inhibited the growth of breast cancer xenograft tumors by regulating the PI3K/AKT signaling pathway and down-regulating the expression of proteins such as WAVE3, p53, p-PI3K, and p-AKT, resulting in a tumor inhibition rate of 59.55%. Ren [38] found that AA exerted anti-tumor effects by decreasing the phosphorylation levels of PI3K, Akt, mTOR, and other enzymes, and it was able to induce the growth cycle of cancer cells to stop at the G0/G1 phase, and the cell survival rate of ovarian cancer was reduced by 50%, and the number of apoptotic cancer cells was increased by 7 to 10-fold after treatment with AA (40 µg·mL^−1^), which fully demonstrated the antitumor potential of AA in ovarian cancer cells. Huang et al. [39] demonstrated that AA (10–40 µM) significantly suppressed the migration and invasive capabilities of Renal Cell Carcinoma (RCC). Through mechanistic investigations, they were the first to identify that AA inhibits RCC proliferation and metastasis by suppressing the ERK1/2 and p38MAPK signaling pathways. It has been reported [40] that AA also induced apoptosis in non-small cell lung cancer cells (A549) by inhibiting COX-2 activity, which in turn down-regulated the PI3K/AKT/mTOR signaling pathway and inhibited the migration of the cancer cells in a concentration-dependent manner, and the 24-h migration rate of AA-treated (15, 30, 60 µM) A549 cells (71.02%, 44.05%, 19.34%) was significantly reduced compared to untreated cells (85.2%). It is worth noting that AA did not show toxicity to healthy lung cells or red blood cells under specific experimental conditions, but this does not mean that it is completely non-cytotoxic. Therefore, more systematic toxicity assessments are needed in the future.

AA also showed significant inhibitory effects on drug-resistant cancer cells. It has been found [41] that AA promotes apoptosis in Doxorubicin-resistant MCF-7 breast cancer cells in a dose-dependent manner, mainly by increasing ROS production, decreasing ATP content in cancer cells, and promoting apoptosis in Doxorubicin-resistant MCF-7 breast cancer cells. Liu et al. [42] first demonstrated that AA prompted apoptosis in cisplatin-resistant nasopharyngeal carcinoma cells by activating p38 MAPK phosphorylation, upregulating the ratio of pro-apoptotic proteins Bax/Bak, and upregulating the expression of Caspase-3, 8, and 9. A study by Zhang [43] found that AA inhibited the proliferation of adriamycin-resistant chronic myeloid leukemia K562/ADR cells and reversed their resistance to adriamycin by down-regulating the expression of MRP1 and P-gp after inhibiting the Wnt/β-catenin signaling pathway. These results highlight the potential of AA to overcome drug resistance, but its applicability in different drug resistance models still needs to be further explored. In addition, whether long-term use of AA will cause cancer cells to develop resistance to AA remains a key question.

Signaling pathways and metabolic dysfunction play crucial roles in disease progression, particularly the transforming growth factor-β (TGF-β) signaling pathway, which exerts key effects in idiopathic pulmonary fibrosis, lung cancer, and other cancers [44]. The synergistic use of AA with other pharmacologies can better exert its antitumor effects. A study by Lian [45] found that AA combined with naringenin inhibited both metastasis and invasion of melanoma and lung cancer cells via the TGF-β-Smad-MMP2 pathway. In mouse melanoma and lung cancer models as well as in vitro studies, it was found that the synergistic use of AA with naringenin could inhibit Smad3-mediated MMP2 transcription, increase TIMP, and enhance Smad7, thereby inhibiting TGF-β/Smad3 signaling, and finally inhibit MMP2 to achieve the antitumor effect. Chen et al. [46] found that the combination of AA with luteolin could enhance the anticervical cancer activity of AA. The combination of AA and luteolin could inhibit the tumor cells in the sub-G1 phase and induced apoptosis mediated by caspases, while the growth and migration of the tumor cells were also inhibited significantly. Mechanistic studies found that luteolin synergistic treatment with AA can inhibit down-regulated PI3K/AKT signaling (PI3K, AKT, and p70S6K), JNK/p38 MAPK signaling and FAK signaling and up-regulate ERK signaling, which can induce apoptosis and inhibit the migration of cancer cells to achieve anti-tumor effects. In addition, another researcher found [47] that AA, by rebalancing Smad7/Smad3, was also able to ameliorate renal fibrosis caused by long-term cisplatin administration in tumor-bearing mice and also enhanced the antitumor effects of cisplatin to some extent. Mechanistic studies have shown that AA promotes tumor cell apoptosis and inhibits cell proliferation by increasing the expression of Caspase-3 and suppressing the expression of Ki-67 and PCNA. Although combination therapy shows promise for AA, there is still no definitive standard for drug interactions or optimal dosage in different tumor models.

Based on the antitumor effects of AA, some researchers have recently predicted the feasibility of AA in the treatment of glioblastoma (GBM), a primary brain tumor, based on a network pharmacology perspective [48]. AKT1, PRKCB, IL-6, TNF and EGFR were identified as key regulatory genes for AA treatment of GBM by SwissTarget and TargetNet databases. Further analysis by protein interaction network and pathway enrichment indicated that AA may intervene in GBM proliferation, angiogenesis, and intervene in the treatment of GBM by inhibiting the molecular functions of the AKT1-PRKCB signaling pathway, modulating EGFR tyrosine kinase resistance, and affecting the binding of tumor necrosis factor receptor. AKT1 and PRKCB have the strongest binding ability to AA and are important proteins for AA to exert anti-tumor effects. The results of the study indicate that AA has potential application value in the treatment of GBM. However, further cellular and animal experiments are needed to assess its safety and efficacy.

Current research findings indicate that the anti-tumor efficacy of AA is not reliant on a single target but rather involves modulating a range of key signaling pathways and their upstream and downstream molecules, thereby establishing a complex regulatory network to exert its anti-tumor effects. AA suppresses a range of downstream pro-metastatic and anti-apoptotic molecules (e.g., VEGF, MMPs, FAK, Cyclins, Bcl-2, MDR-transporters) by downregulating the expression of core signaling pathways (e.g., PI3K/AKT/mTOR, ERK MAPK, TGF-β/Smad, Wnt/β-catenin, etc.) and upregulating the expression and activity of pro-apoptotic molecules (e.g., Bax/Bak, Caspases), which regulate the cell cycle and induce apoptosis. These upstream and downstream regulatory mechanisms interact synergistically, forming a complex network that underpins the multi-target and multi-pathway anti-tumor effects of AA. In this manner, it exerts inhibitory effects on a range of malignant tumors, including breast, lung, ovarian, and nasopharyngeal cancers. However, current research still has certain limitations. First, studies on the antitumor effects of AA involve multiple models (in vivo/in vitro, cell/animal), resulting in a relatively wide range of effective doses for AA. Therefore, future research should focus on standardized models and dose–response relationships. In addition, AA’s antitumor effects involve multiple signaling pathways, but research on each pathway is relatively independent. The primary and secondary roles of each pathway, their relationships, and whether they have a coordinated effect require further study. The main mechanism of action is shown in Table 2.

### 2.4. Hypoglycemic Effects

AA has also shown some therapeutic potential in metabolic diseases in recent years. In a streptozotocin (STZ)-induced diabetic rat model [57], AA significantly lowered blood glucose levels and promoted the survival and proliferation of pancreatic β-cells through activation of the Akt signaling pathway and up-regulation of the expression of the anti-apoptotic protein, Bcl-xL, in an attempt to ameliorate the effects of pancreatic β-cell damage or dysfunction in type I diabetes. Ji [58] and his colleagues found that AA (10, 30 mg·kg^−1^) significantly reduced blood glucose levels as well as sCr levels and BUN levels in STZ-induced diabetic rats, and ameliorated pathological injuries such as vacuolization of renal tubular epithelial cells and cell detachment from the lumen of renal tubules in diabetic nephropathy rats.

Impaired insulin secretion by pancreatic β-cells is a prominent feature of type II diabetes mellitus [59]. For type II diabetes [60], AA can increase β-cell maturation by modulating the TNF-α/Mfn2 signaling pathway as well as up-regulating the expression of Mfn2 and Ucn3 proteins to achieve hypoglycemia. In addition, AA can effectively inhibit adipogenesis by down-regulating the expression of adipogenesis-related genes (e.g., PPARγ, SREBP-1c, and FAS), while promoting energy expenditure through up-regulation of Uncoupling Protein 2. In Wang’s [61] study on an animal model of spontaneous type II diabetes, it was found that AA (25 mg·kg^−1^) treatment attenuated fasting blood glucose levels in GK rats, and serum insulin levels were also reduced by 70% compared to the control group, which ameliorated insulin resistance to some extent. In addition to lowering blood glucose, Wang also found that AA was able to protect beta cells by inhibiting pancreatic fibrosis and exerting a hypoglycemic effect. In summary, AA reduces blood glucose, protects β cells, and regulates lipid metabolism through potential multi-target mechanisms in specific models. These results provide some theoretical support for exploring AA as a potential strategy for treating diabetes, but animal models cannot fully simulate the complexity of human diabetes. In addition, whether AA can become a safe and effective hypoglycemic drug should also be compared with existing hypoglycemic drugs (such as metformin) and verified through extensive safety experiments.

### 2.5. Neuroprotective Effects

AA has been reported to be effective in improving neurological disorders caused by aluminum toxicity, Alzheimer’s disease (AD), epilepsy, and other disorders, and can also alleviate hippocampal neurodegeneration and memory deficits [62]. Some researchers found [63] that AA was effective in reversing the abnormal levels of acetylcholinesterase and oxidative stress in the hippocampus induced by aluminum intoxication in rats and alleviated memory deficits in rats by increasing the hippocampal cell density. AD is a progressive developmental neurodegenerative disease that is one of the leading causes of dementia [64]. Researchers found [65] that AA exhibited significant neuroprotective potential in an in vitro model of AD. After AA (10 nM) intervention, ROS production was significantly decreased, mitochondrial structural changes were inhibited, expression of caspase-9 and caspase-3 was decreased by 60%, and Bax/Bcl-2 ratio was reduced (from 2.5 to 0.8) in AD rat cells, inhibiting apoptosis. Amyloid beta (Aβ) peptide causes neurotoxicity in Alzheimer’s disease and is an important pathogenic protein in AD [66]. Cheng et al. [67] explored the protective effect of AA against Aβ_23–25_-induced neurotoxicity in neurally differentiated PC12 cells. AA was found to reduce apoptosis by attenuating mitochondrial dysfunction and indirectly attenuate neurological damage by reducing inflammatory responses through inhibition of the NF-κB signaling pathway. This study also showed that AA protects PC12 cells from Aβ_23–25_-induced apoptosis as well as hyperphosphorylation of tau through activation of the PI3K/Akt/GSK-3β signaling pathway thereby protecting the cells from neurological damage. In a 2025 study, Varada et al. [68] found that AA can alleviate cognitive deficits caused by excessive Aβ expression in 5xFAD mice. AA can increase the expression of the antioxidant regulatory transcription factor NRF2 and antioxidant genes in the mouse brain, thereby activating antioxidant activity and alleviating cognitive impairment in mice by improving mitochondrial function. Importantly, the study found that the cognitive improvement effect of AA was independent of changes in Aβ plaque burden, suggesting that it may act on Aβ downstream pathways or parallel pathways, providing new insights for the treatment of late-stage AD.

Another study [69] demonstrated that, for cognitive deficits caused by epilepsy, injection of AA (15 mg·kg^−1^) significantly improved kainic acid-induced seizure, and further studies revealed that the mode of action was mainly through the enhancement of AKT activity, as well as the inhibition of calpain expression, which protects the neuronal cells from hippocampal damage and maintain cognitive function. He et al. [70] constructed a neonatal rat spinal cord neurotoxicity model using ropivacaine, which was used to explore the effects of AA on neurotoxicity. The results showed that AA dose-dependently reduced the rate of apoptosis and inflammatory cell infiltration in rat spinal cord tissues. Mechanistic studies showed that AA was able to elevate cAMP content, PKA mRNA and protein expression, p-CREB phosphorylation, and BDNF protein expression in rat spinal cord tissues, suggesting that AA attenuates ropivacaine-induced neurotoxicity in neonatal rats through the c AMP/PKA signaling pathway. A recent study by Hu [71] found that AA was able to exert neuroprotective effects by targeting Acyl Coenzyme A Oxidase 1 to inhibit the ferroptosis of neurons in a dose-dependent manner to alleviate brain damage in subarachnoid hemorrhage (SAH) mice. By comparison with mice in the SAH group, it was found that AA treatment was able to increase the number of neurons in SAH mice. Mechanistic studies showed that AA treatment could reverse the down-regulation of ACOX1 expression caused by SAH, restore GPX4 activity and GSH levels, and reduce MDA production, and thus inhibit lipid peroxidation as well as ferroptosis of neurons.

In summary, AA has demonstrated good neuroprotective effects in various models of neurological diseases. In addition, the mechanism of action of AA varies across different studies. Its core mechanism of action lies in effectively alleviating oxidative stress, improving mitochondrial dysfunction, and inhibiting various forms of cell death. Therefore, it achieves neuroprotection through multi-target, multi-pathway synergy. Nevertheless, in order to successfully transition from the experimental stage to clinical application, it is still necessary to overcome the limitations of the model and clarify the pharmacokinetic characteristics and safety of AA in the human body.

### 2.6. Cardioprotective Effects

AA has great potential in preventing cardiovascular diseases: it can improve doxorubicin-induced cardiac output abnormalities by blocking the release of cTnI from plasma, and it can also ameliorate oxidative damage by activating the AKT and Nrf2 signaling pathways to improve cardiac function [72]. Myocardial ischemia–reperfusion injury (MIRI) is the main cause of heart failure in patients with coronary artery disease [73]. It has been found [74] that AA can alleviate MIRI by activating the Akt/GSK-3β/HIF-1α signaling pathway, regulating glucose metabolism, resisting oxidative damage, protecting mitochondria, and regulating mitochondrial autophagy, among other pathways. Mechanistic studies suggest that another pathway by which AA ameliorates MIRI may be through activation of the p38 MAPK and Bcl-2 signaling pathways, which reduces cellular oxidative stress and p38 MARK phosphorylation and attenuates cardiomyocyte autophagy. Other studies have found [75,76] that AA exerts cardioprotective effects through a combination of antioxidant and anti-apoptotic pathways. In terms of antioxidants, AA was able to scavenge ROS, restore mitochondrial redox balance, and reduce ROS accumulation in myocardial tissues and cells. In terms of anti-apoptosis, AA inhibits mitochondrial apoptosis by suppressing MAPK phosphorylation, up-regulating the anti-apoptotic protein Bcl-2, and down-regulating the pro-apoptotic protein Bax, balancing the Bcl-2/Bax ratio, and inhibiting the activation of caspase-3,9. This confirms that AA attenuates oxidative stress and apoptosis in MIRI by targeting the ROS-MAPK-mitochondrial apoptotic pathway. Qiu et al. [77] showed that AA could attenuate ischemic myocardial injury (IMI) by modulating mitophagy and Glucophage-based. In oxygen-glucose deprivation (OGD)-treated cardiomyocytes, AA (10 µM) significantly increased cell viability and inhibited apoptosis. In in vivo experiments, AA (25 mg·kg^−1^·day^−1^) significantly reduced the area of myocardial infarction and improved cardiac function in mice. Mechanistic studies have shown that AA dually regulates mitochondrial and glucose metabolism through activation of the AMPK signaling pathway and the PI3K/Akt signaling pathway for the treatment of ischemic heart disease.

Aarti Tiwari [78] demonstrated that AA could prevent hyperlipidemia to some extent and reduced cardiovascular diseases such as atherosclerosis, myocardial infarction, heart disease, and stroke induced by hyperlipidemia. The experimental results showed that the serum levels of total cholesterol (TC) and triglyceride (TG) were decreased in rats orally administered with AA (10, 20 mg·kg^−1^) compared with the control group of hyperlipidemic rats, and the inhibitory effect was more significant in the group orally administered with 20 mg·kg^−1^ AA, with the decrease of TC as well as TG by 2.34 and 4.44 times, respectively. In addition, the atherogenic index was significantly reduced in the administered group after oral administration of AA. Mechanism of action studies have shown that AA lowers cholesterol by inhibiting HMG-CoA reductase. AA can reduce the oxidative stress caused by hyperlipidemia in the liver and heart on the basis of reducing hyperlipidemia, thus reducing the probability of cardiovascular disease, and the ability of AA to reduce oxidative stress may be related to its antioxidant activity [79].

### 2.7. Antibacterial Effects

AA has significant antibacterial activity against Gram-positive bacteria. Research has found that AA can inhibit 19 different strains of Clostridium difficile to varying degrees at low concentrations (10–20 µg·mL^−1^) [80]. The results showed that AA can interfere with the integrity of bacterial cell membranes, causing membrane potential loss and leakage of intracellular substances, reducing colony size and inhibiting bacterial growth. By inhibiting the GlgE target in bacteria, AA hinders normal glycogen synthesis, which is another way it slows down bacterial growth [81]. Kandaswamy [82] found that AA (MIC = 20 µg·mL^−1^) had an inhibition rate of 89.66% against methicillin-resistant *Staphylococcus aureus* (MRSA) and reduced bacterial biofilm biomass by 85.66%, effectively inhibiting biofilm formation and exhibiting significant antimicrobial activity.

AA can also interact with DNA or proteins formed by the mitotic spindle, affecting cell replication and interfering with cell division. It exhibits inhibitory activity against various Gram-negative bacteria (urinary tract pathogenic *Escherichia coli* CFT073, *Enterobacter cloacae* ATCC BAA-2468, *Pseudomonas aeruginosa* ATCC 25000) (MIC values of 1536, 1024 and 1536 µg·mL^−1^, respectively) [83]. Additionally, against *Shigella*, AA can activate the TLR pathway and downstream MAPK signaling pathway, thereby increasing the phosphorylation levels of three key MAPKs—ERK, p38, and JNK. This leads to a significant upregulation in the transcription levels of antimicrobial peptide genes, ultimately causing secreted antimicrobial peptides to disrupt the integrity of bacterial cell membranes and result in bacterial death. Thus, AA can inhibit bacterial growth by regulating the MAPK/TLR pathway. [84]. It can thus be seen that AA achieves multi-target antibacterial action by disrupting membrane integrity, inhibiting glycogen metabolism (GlgE), and interfering with cell division.

In addition to bacteria, AA also exhibits activity against fungi. Wang et al. [85] found that AA effectively inhibited *Candida albicans* activity both in vivo and in vitro. When used alone, AA inhibited the accumulation of ROS in *Candida albicans* CA10 cells. In vitro antifungal experiments showed that when used in combination with azole antifungal drugs (such as fluconazole), AA reduced the MIC of *Candida albicans* by 4–8 times (from 64–128 µg·mL^−1^ to 16–32 µg·mL^−1^). In a wax moth larvae infection model, it was shown that combining fluconazole with AA increased the antifungal efficacy from 45% to 70% and reduced melanin nodules (a marker of fungal load) in the infected larvae tissue while inhibiting hyphal growth. Currently, there is limited research on the antibacterial mechanism of AA. However, existing studies have confirmed that the antibacterial activity of AA exhibits a certain degree of strain dependency. The MIC (20 µg·mL^−1^) of AA against Gram-positive bacteria (such as MRSA) is significantly lower than that against Gram-negative bacteria (MIC > 1000 µg·mL^−1^). The outer membrane is a core characteristic distinguishing Gram-negative from Gram-positive bacteria, as it impedes the penetration of antibacterial compounds into cells. This may explain why AA exhibits reduced inhibitory activity against Gram-negative bacteria. AA exhibits synergistic effects with antibiotics. In vitro antimicrobial experiments have shown that when used in combination with azole antifungal drugs (such as fluconazole), AA reduces the MIC of *Candida albicans* [85]. AA also demonstrates significant synergistic effects with antibiotics in combating Gram-positive bacteria, exhibiting considerable potential for clinical application. Although AA has a significant inhibitory effect on Gram-positive bacteria, its inhibitory activity against Gram-negative bacteria is relatively weak, its penetration mechanism into biofilms remains unclear, and current research is largely limited to in vitro experiments. These factors constitute the primary bottlenecks hindering its widespread application.

### 2.8. Protective Effect on the Skin and Wound Healing Effect

AA, as a natural product, is an effective therapeutic agent for wound healing [86]. AA has been proven to have a wide range of applications in skin care products such as anti-aging, skin whitening, and sunscreen, and has demonstrated efficacy in the treatment of a variety of dermatological conditions [87,88]. In terms of inflammatory skin diseases, it has been confirmed [89] that oral administration of high-dose AA (100 mg·kg^−1^) can effectively ameliorate skin damage induced by imiquimod, reduce erythema, desquamation, and thickening of the skin, as well as significantly reduce mast cell infiltration, protect skin integrity, and also protect the skin integrity by inhibiting the abnormal elevation of the pro-inflammatory factors IL-17A and IL-23 in the serum to alleviate psoriasis in mice. Liu [90] found that quercetin, the active ingredient in *C. asiatica*, likewise exerts antipsoriasis effects by reducing IL-17A secretion from inflammatory cells through mediating STAT3 phosphorylation to inhibit the IL-23/IL-17A inflammatory axis. In the future, it could be further explored whether quercetin could be used synergistically with AA thereby enhancing the antipsoriasis effect of AA. At the same time, the safety and efficacy of long-term use of high doses of AA in humans still needs to be evaluated.

The protective effect of AA is also reflected in its ability to counteract external damaging factors. For example, it can inhibit the generation of ROS and lipid peroxidation induced by ultraviolet A (UVA) rays, and downregulate the expression of MMPs and p53, thereby providing a certain degree of protection for the skin [91]. Some scholars have found that AA can effectively promote the proliferation of fibroblasts and collagen synthesis, and treat keloids by activating the PPAR-γ pathway and inhibiting TGF-β1-induced expression of collagen and PAI-1 in fibroblasts [92,93]. However, the treatment of keloids presents certain challenges, and further research is needed to confirm whether this treatment alone can effectively treat keloid scars. In addition, AA increases the tensile strength of the skin and indirectly promotes wound healing through antifungal effects [94,95]. Wang [96] found that the expression of MMP-1, MMP-8, MMP-9, caspase-3 and caspase-8 in human skin keratinized cells (HaCaT) was inhibited and wound healing was accelerated after AA intervention. AA was also found to indirectly promote tissue regeneration by inhibiting the expression of RAGE, p38, and JNK and exerting antioxidant and anti-inflammatory activities. Liu’s recent study [97] demonstrated that AA was able to attenuate Nitrogen mustard (NM)-induced skin damage by inhibiting ERS. By exploring the effects of AA on NM-induced HaCaT cells, it was found that NM was able to reduce cell viability disrupt cell morphology, and induce apoptosis, as well as inducing the secretion of TNF-α and IL-6 and enhancing the inflammatory response, and that AA pretreatment was able to reverse these problems to a certain extent. This study further revealed that AA also inhibited ERS to achieve cytoprotection by down-regulating the expression of GRP78, a biomarker of NM exposure-induced HaCaT cells, and inhibiting the IRE1-XBP1 and ATF6 signaling pathways, further demonstrating that AA prevented NM-induced skin damage by inhibiting ERS. AA participates in one or more phases of the skin repair process, showing active roles in tissue regeneration, cell migration and wound repair processes [98]. In summary, AA demonstrates potential for protecting the skin and promoting wound healing by influencing multiple pathways, including inflammation, oxidative stress, and cell proliferation/apoptosis, providing a solid scientific basis for the development of highly effective, low-toxicity skin protection drugs.

The signaling pathways associated with the diverse pharmacological effects of AA are illustrated in Figure 2.

## 3. Effect of Structural Modification of Asiatic Acid on Pharmacological Effects

Currently, the research on the biological functions of AA and its potential mechanism of action is quite extensive, but due to the drawbacks of its poor water solubility and low oral bioavailability, the potential impact of some of the efficacy of AA has been limited to a certain extent. In recent years, the enhancement of their pharmacological properties through structural modification strategies has become a hot research topic. As demonstrated in the relevant literature, the structural modifications of AA are primarily concentrated at positions C-2, C-3, C-11, C-23, and C-28 [4,5,99,100]. For example, the introduction of an α,β unsaturated ketone structure at the C-11 site of the A ring, or the introduction of an aniline at the C-28 site and linking it through an amide bond significantly enhanced the in vitro antitumor activity of AA [5]. These modification strategies provide a theoretical basis for enhancing the biological activity of AA and provide ideas and insights for the preparation of novel AA derivatives.

### 3.1. Improvement of Anti-Tumor Effect

**Figure 3:**
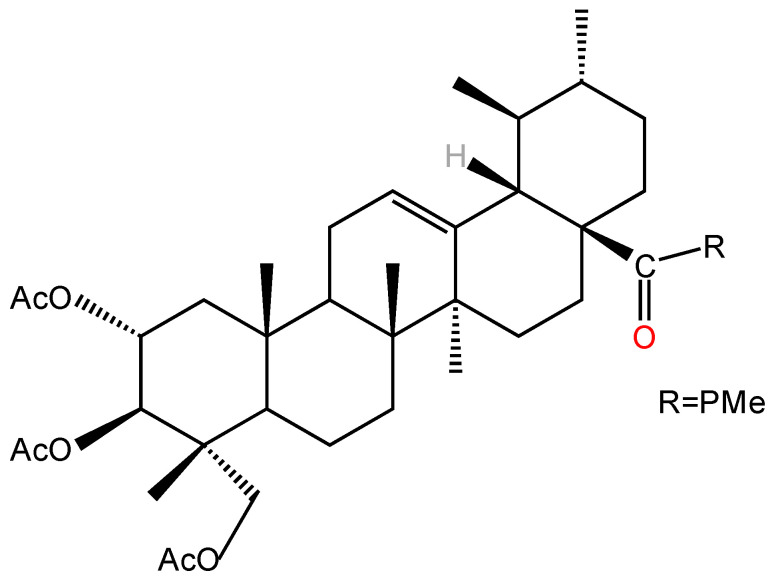
Chemical structural formula of N-(2α,3β,23-acetoxyurs-12-en-28-oyl)-l-proline methyl ester (AA-PMe).

Structural modifications significantly enhanced the cytotoxicity and selectivity of AA significantly enhanced by structural modifications. Mei [101] found that ring-splicing of A with a nitrogen-containing heterocyclic ring and the introduction of an aromatic amide at its C-28 increased the antitumor activity of AA, and all similar derivatives had higher antitumor activity than the parent compound, AA, against both SGC7901 and A549 cell lines. Jing et al. [99] synthesized 15 semi-synthetic derivatives by chemically modifying the C-2, C-3, C-23 hydroxyl (acetylation) and C-28 carboxyl groups (amino acid ester substitution) of AA and systematically evaluated their antitumor and antiangiogenic activities. In vitro antitumor assays revealed that the derivative N-(2α,3β,23-acetoxyurs-12-en-28-oyl)-l-proline methyl ester (AA-PMe) (Figure 3) with acetylation at C-2, 3, and 23 positions and introduction of an amino acid ester group at C-28 position had significantly higher antitumor activity than that of AA, and the IC_50_ of AA-PMe was significantly lower against tumor cells (A549, HepG2) with IC_50_ as low as 0.3–4.8 µM, which is a significant improvement over AA (IC_50_ = 4.0–55.1 µM). This study also found that derivatives with only modification of the amino acid at the C-28 position or acetylation of the hydroxyl group alone were less active, further suggesting that the synergistic effect of C-28 esterification and polyhydroxy acetylation is an important factor in the enhancement of AA activity. The stability experiments proved that AA-PMe has good stability and does not denature at 37 °C and −20 °C, which provides a basis for the subsequent development of new dosage forms. In further studies, Jing et al. [102] showed that AA-PMe could inhibit cancer cell proliferation by down-regulating the cell cycle regulatory proteins cyclin D1 and CDK4 and inducing cancer cells to block at the G0/G1 phase. Meanwhile, AA-PMe could also significantly inhibit the migration and invasion of gastric cancer cells by inhibiting the expression of matrix metalloproteinases MMP-2 and MMP-9 and decreasing the degradation ability of the extracellular matrix. Compared with AA, AA-PMe has more significant advantages in pharmacological activity. The IC_50_ of AA-PMe against SGC7901 and HGC27 was 4.48 µM and 7.92 µM, respectively, which was 8–9-fold lower than that of AA (36.9 µM and 61.23 µM), demonstrating stronger antigastric cancer activity. Another study showed that the core mechanism of AA-PMe anti-gastric cancer lies in the inhibition of Signal Transducer and Activator of Transcription 3 (STAT3) signaling pathway, which blocks the tyrosine phosphorylation of STAT3 by inhibiting the activity of JAK2 kinase, down-regulates the expression of cyclin D1, c-Myc, and Bcl-2 expression and up-regulating the pro-apoptotic protein Bax, reducing the proliferation of gastric cancer cells and their mobility, and thus inducing apoptosis to achieve anti-tumor effects [103]. In addition, AA-PMe showed a much lower lethal sensitivity to zebrafish embryos, demonstrating its favorable safety [6].

**Figure 4:**
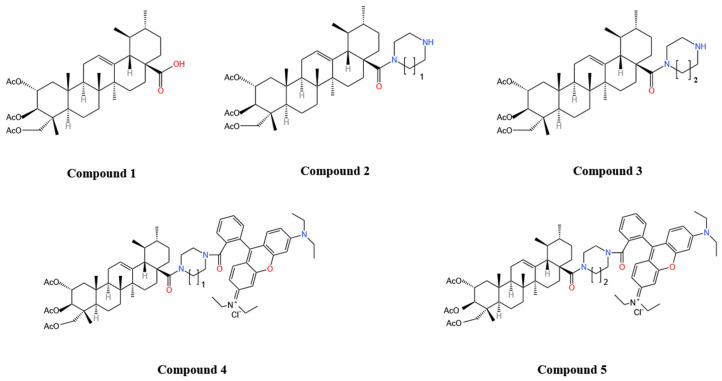
Chemical structural formulae of compounds **1**–**5**. Compound **1**: (2α,3β,4α,6β) 2,3,23-Tris(acetyloxy)-urs-12-en-28-oic acid, Compound **2**: (2α,3β,4α)-2,3,23-Tris(acetyloxy)-N-(piperazinyl)-urs-12-en-28-amide, Compound **3**: (2α,3β,4α)-2,3,23-Tris(acetyloxy)-N-(homopiperazinyl)-urs-12-en-28-amide, Compound **4**: N-{6-diethylamino-9-[2-({4[(2α,3β,4α)-2,3,23-tris(acetyloxy)-28-oxours-12-en-28-yl)-1-piperazinyl}carbonyl]phenyl]-3H-xanthen-3-ylidene)-N-ethylethanaminium chloride, Compound **5**: N-{6-diethylamino-9-[2-({4[ (2α,3β,4α)-2,3,23-tris(acetyloxy)-28-oxours-12-en-28-yl)-1,4-diazepan-1-yl}carbonyl]phenyl]-3H-xanthen-3-ylidene)-N-ethylethanaminium chloride.

Some researchers have found [104] that attaching rhodamine B molecules to triterpene acids (e.g., ursolic, oleanolic, betulinic, maslinic acid, asiatic acid) significantly increases cytotoxicity, and that with similar structural modifications, maslinic acid derivatives (EC_50_ = 7 nM) and AA derivatives were the most cytotoxic to A2780, and the introduction of acetyl groups at the C-2 and C-3 positions was necessary to make the derivatives selective for tumor cells [105]. Kraft [3] synthesized 6 AA derivatives based on AA (Figure 4). Among them, compound 7 (acetylated homopiperazinyl rhodamine B coupling) exhibited the best biotoxicity with significant cytotoxicity against A2780 ovarian cancer cells (EC_50_ = 0.8 nM), a very significant enhancement over AA (28.2 ± 0.3 µM) and maslinic acid derivatives, this may be due to the fact that the asiatic acid derivatives have an additional acetyl group at the C-23 position that enhances their solubility. In addition, compound 5 remained highly cytotoxic (IC_50_ ≈ 2.7–11.7 nM) in 3D tumor spheroid models, effectively inhibited sphere growth, and was free of cross-resistance in rectal and breast cancer resistance models. Mechanistic studies have shown that compound 5 triggers apoptosis by rapidly disrupting cellular metabolism, which is very different from the mode of action of conventional chemotherapeutic drugs that interfere with DNA synthesis and repair [106], interfere with protein synthesis, and inhibit cell division [107]. Lu et al. [4] first modified the carboxyl group at the C-23 site of AA, and found that the C-23-amide derivative was able to immobilize the hydrogen atoms on the amide on the AA backbone through intramolecular hydrogen bonding and enhance its water solubility and membrane permeability, thus improving bioavailability, and that the derivative exhibited some cytotoxicity against numerous cancer cells, with the strongest effect on HL-60 cancer cells (IC_50_ = 0.47 µM), exhibiting some selectivity.

**Figure 5:**
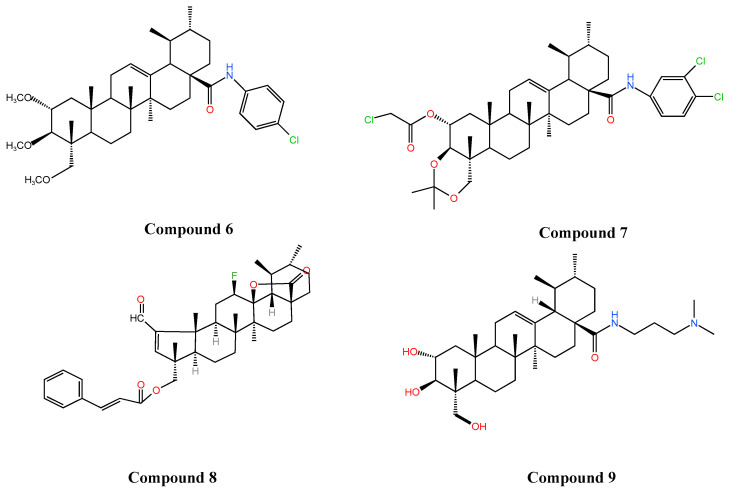
Chemical structural formulae of compounds **6**–**9**.

Studies have found that substitution at the C-28 position enhances the antitumor activity of AA and that different substitutions affect the mode of action to varying degrees [108]. Li et al. [5] showed that the introduction of amino and carbonyl groups at the C-28 and C-11 positions of AA, respectively, significantly increased the anti-HepG2 cell activity of AA (IC_50_ 5.97 µM, 8.89 µM, respectively), which was 4–6-fold higher than that of AA (IC_50_ = 34.9 µM). Wang and colleagues [109,110] synthesized new AA derivatives (Compound **6**, Compound **7**) by introducing active groups at C-28 position and assayed the antitumor activity by MTT assay, which showed that the IC_50_ of Compound **6** and Compound **7** (Figure 5) against HepG2 and SGC7901 cell lines were 15.44 µM, 17.53 µM and 9.39 µM, 7.08 µM against HepG2 and SGC7901 cell lines, respectively, which were significantly higher than that of AA (IC_50_ > 50 µM for both cell types), and the antitumor effects were obviously enhanced. Other reports have indicated that fluorine substitution effectively improves the lipid solubility, membrane permeability, binding affinity, and stability of AA [111], and also enhances its antitumor activity [112]. Fluorinated AA derivatives showed significantly higher antiproliferative activity (IC_50_ of 0.67 µM, 0.71 µM, respectively) against both cervical (HeLa) and colon (HT-29) cancer cell lines compared to AA (IC_50_ of 52.47 µM, 62.30 µM). Mechanistic studies showed that Compound **8** (Figure 5) was able to block the cell cycle at the G0/G1 phase by up-regulating the expression of the cycle regulatory proteins p21cip1/waf1 and p27kip1, as well as down-regulating the expression of cyclin E and cyclin D3, and promote apoptosis by inhibiting the expression of Bcl2 and promoting the expression of Bax proteins, to achieve an anti-tumor effect [112]. Wang et al. [7] found that some other AA derivatives exerted anti-tumor effects by down-regulating the expression of the Ras/Raf/MEK/ERK pathway and blocking the cell cycle at the G1/S and G2/M phases. In summary, AA derivatives have achieved significant enhancement in antitumor activity, diversity of mechanism of action, and selectivity through precise structural modification, providing a direction of choice for the development of novel anticancer drugs.

### 3.2. Improvement of Hypoglycemic Effect

Glycogen phosphorylase (GP) is usually associated with the development of type II diabetes mellitus [113], and the incidence of diabetes mellitus can be effectively reduced by inhibiting the activity of GP, and GP inhibitors are currently shown to have favorable hypoglycemic effects in animal model experiments and clinical practice [114]. It has been reported [115] that AA, a major component of the traditional Chinese herb *C. asiatica*, has been shown to be a natural GP inhibitor. The enhanced efficacy of AA derivatives in anti-GP activity has also been verified, Zhang et al. [116], who structurally optimized AA by esterification modification of the C-28 site of AA, demonstrated a 4-fold increase in anti-GP activity (IC_50_ = 3.8 µM) compared to AA (IC_50_ = 17 µM). And combined with the conformational relationship analysis, it was found that asiatic acid benzyl ester, a new derivative of AA with 2α-OH, exhibited similar effects as GP inhibitors, and the inhibitory activity was also significantly higher than that of AA. Specific esterification modification of AA derivatives substantially enhances GP inhibitory activity, providing an important direction for the development of novel antidiabetic drugs. In another study on human carbonic anhydrase VA (hCA VA) [117], AA derivatives were modified by sulfonylation to enhance their inhibitory activity and selectivity against hCA VA. One of the trisulfonated AA derivatives showed a Ki value of 36.2 nM and a selectivity of 144.5 nM for hCA VA, which was significantly higher than that of the clinical standard drug acetazolamide (63.0 nM). In addition, the water solubility of this derivative was enhanced by 20-fold compared to AA, solving the problem of poor solubility of natural triterpenoids. Trisulfonated AA derivatives have great potential as potent and highly selective hCA-VA inhibitors for the treatment of obesity and other related diseases.

### 3.3. Improvement of Neuroprotective Effect

In recent years, significant progress has been made in harnessing the neuroprotective effects of AA and its derivatives. It was shown [118] that derivatives (e.g., AS-2, AS-2-9-006, and AS-9-006) obtained by structural modification of the C-2 and C-28 sites of AA exhibited significant advantages over AA in improving cognitive function and neuroprotection. These derivatives significantly reversed scopolamine-induced memory impairment at low doses (1–10 mg·kg^−1^). In the evasion test, AS-2, AS-2-9-006, and AS-9-006 (10 mg·kg^−1^) improved retention times to 163.7, 165.6, and 154.4 s, respectively, which were significantly higher than 66.3 s for AA. In addition, these derivatives also shortened the escape latency and simplified the swimming path in mice in a water maze test, significantly improving memory impairment. Mechanistic studies have shown that such derivatives work by improving the synthesis of acetylcholine to enhance cognitive performance, which in turn exerts a preventive and therapeutic effect on Alzheimer’s disease. Jew [100] et al. hypothesized that the free carboxyl group is a key factor in the ability of AA to exert neuroprotection, and through their study, they found that AA derivatives containing a free carboxyl group at the C-28 locus were more neuroprotective in vitro, with a neuroprotection rate as high as 97%.

### 3.4. Other Effects

Periodontitis is a chronic bacterial infection that can lead to tooth loss [119]. AA derivatives also have a positive role in promoting differentiation of human periodontal ligament stem cells (hPDLSCs). Thamnium et al. [120] substituted the carboxyl group on AA with dimethylaminopropylamine to form a new derivative that improved the poor water solubility and low potency of AA and enhanced its biological effects on type I collagen synthesis and induction of osteogenesis in stem cells. Mechanistic studies revealed that Compound **9** (Figure 5) upregulated the expression of BMP2 through the Erk signaling pathway and promoted the differentiation of hPDLSCs, thereby increasing the osteogenic activity of hPDLSCs and the healing activity significantly. Sumrejkanchanakij [121] prepared a new AA derivative (asiatic acid methyl ester) by esterifying AA. Asiatic acid methyl ester was found to be more cytotoxic and could stimulate osteogenic differentiation of hPDLSCs at a low concentration (2.5 µM) by enhancing WNT3A expression through activation of Wnt signaling as well as β-catenin nuclear translocation. AA derivatives show great potential in periodontal regeneration and are expected to become novel therapeutic agents for periodontal regeneration and reduce the cost of treatment. The structural modification of AA not only significantly improves its solubility and stability, but also greatly enhances its pharmacological activities in anti-tumor, anti-diabetes, neuroprotection and osteogenic induction. Table 3 summarizes the main modification strategies of AA derivatives and their effects on the efficacy in recent years.

Compound **6**: N-(2, 3, 23-trimethoxy-ursane-12-ene-28-carbonyl)-p-chloroaniline; Compound **7**: N-(2α-chloroacetyl-3β,23-O-isopropylidene-ursane-12-ene-28-carbonyl)-3,4-dichloroaniline; Compound **8**:2-Formyl-12β-fluoro-23-cinnamoxy-A(1)-norurs-2-en-13,28β-olide; Compound **9**: 2α,3β,23-Tris(acetyloxy)-urs-12-en-28-oic acid

The structural modifications of AA are mainly concentrated at the C-2, C-3, C-23, and C-28 sites, among which C-2, C-3, and C-23 are mainly modified through acetylation, which primarily affects the water solubility and stability of the derivatives. Structural modifications at the C-28 position (such as esterification, rhodamine B coupling, amidation, or sulfonylation) play a key role in enhancing pharmacological activity. Additionally, the size of the compound group introduced at the C-28 position also affects the pharmacological activity of the derivative. Structural modification of AA precisely regulates the water solubility of the molecule, significantly enhancing its pharmacological activity and bioavailability. However, the modified derivatives still pose certain toxicity risks and synthetic complexity issues. In the future, combining novel drug delivery systems could overcome existing bottlenecks and promote the clinical translation of AA derivatives.

## 4. Toxicity Assessment of Asiatic Acid

Preclinical safety and toxicology evaluation, as a critical initial step in the drug development process for new molecules, not only provides a scientific basis for the preliminary screening of candidate compounds but also establishes an essential foundation for their subsequent advancement to the clinical trial stage. Currently, toxicological studies on AA are limited, primarily focusing on *C. asiatica* extracts. Toxicity assessments of *C. asiatica* extracts can indirectly indicate the safety profile of AA. In acute toxicity studies, *C. asiatica* extract was administered at doses up to 10 g·kg^−1^, and no toxic effects were observed in rats. In a chronic toxicity study, Wistar rats were orally administered up to 1200 mg·kg^−1^·day^−1^ of *C. asiatica* extract for six months, and no significant adverse effects or organ damage were noted [122]. Another study demonstrated that the LD_50_ of an ethanolic extract of *C. asiatica* was greater than 2000 mg·kg^−1^ in albino mice, with no fatalities observed in either acute or subacute toxicity studies. Moreover, during the evaluation period, none of the mice exhibited clinical symptoms of poisoning (no abnormalities were observed in behavior, body weight, blood biochemistry, histopathology, or other aspects) [123]. Studies have also reported that in albino rats, continuous administration at doses of 500 and 1000 mg·kg^−1^ for one month did not show toxicity [124]. In a reproductive toxicity study, three doses of ethanolic extracts of *C. asiatica* (100 mg·kg^−1^, 200 mg·kg^−1^, 300 mg·kg^−1^) significantly decreased the number of spermatozoa in the lumen of rat seminiferous tubules compared to the control group, and all doses resulted in reduced or non-progressive sperm motility in rats. Yen [125] investigated the genotoxicity of *C. asiatica* extracts using *Salmonella* typhimurium strains TA98 and TA100. The results demonstrated that *C. asiatica* extracts did not exhibit direct mutagenic effects at lower conventional consumption doses, and the Ames test yielded negative results. The in vitro micronucleus assay of *C. asiatica* extract demonstrated [126] that it did not exhibit significant genotoxicity within the concentration range of 2.25–9 µg·mL^−1^. In a subchronic oral toxicity assay of aqueous extracts of *C. asiatica* [127], administration of 800 mg·kg^−1^·day^−1^ to mice did not result in mortality or observable clinical signs of toxicity. However, an increase in the total number of leukocytes and lymphocytes was noted in both male and female mice during treatment with the aqueous extracts, indicating a potential risk. Acute toxicity studies of AA conducted by Guo [128] demonstrated that zebrafish embryos exposed to 50 µM AA for 24 h began to exhibit mortality. The LC_50_ value of AA was determined to be 49.849 µM, indicating high toxicity. Pericardial edema was observed in zebrafish following treatment with high concentrations of AA, with an edema incidence rate of 10%. Additionally, subacute toxicity studies revealed that prolonged exposure to 30 µM AA significantly increased ROS levels and downregulated the expression of antioxidant enzymes in zebrafish cells, thereby exacerbating oxidative stress-induced damage and leading to apoptosis. The antitumor activity of AA-PMe, a derivative of AA, is significantly enhanced; however, its safe dosage range is narrow, with acute lethality observed at high dose and chronic toxicity noted at low dose, leading to peritoneal fluid accumulation and pericardial effusion in zebrafish [6].

Numerous experiments have demonstrated that *C. asiatica* extract exhibits a relatively favorable safety profile. However, as the primary component of *C. asiatica* acid extract, toxicological studies on AA remain relatively limited, and there is currently a lack of specialized papers or information dedicated to the assessment of its safety and toxicity. In the future, further investigation into the toxicity of AA and its derivatives could provide more comprehensive data to facilitate its transition from basic research to clinical application.

## 5. Advances in Novel Delivery Systems for Asiatic Acid

In vivo and in vitro experiments have revealed that AA has significant efficacy in anti-tumor, anti-inflammatory and neuroprotection, but its low water solubility, rapid degradation, short half-life, low bioavailability, and difficulty in crossing the blood–brain barrier have limited its oral absorption and clinical application [129]. The current application of new dosage forms improves some of the shortcomings of such drugs and points the way to the development of new drugs.

### 5.1. Nanoparticles

Nanoparticles are characterized by small particle size (10–1000 nm) and large specific surface area, which can improve drug bioavailability by improving solubility, permeability and prolonging the release time of the drug as well as its retention time in the intestinal tract [130,131]. The use of nano-formulations for drug delivery offers greater safety and stability, and the approach now has great potential to overcome pharmacokinetic and biopharmaceutical limitations of drugs [132].

Zhang’s team [133] prepared AA-loaded nanoparticles using the solvent evaporation method. The nanoparticles consist of asiatic acid loaded onto chitosan-deoxycholic acid-mannose/biotin-TPGS nanoparticles (CDM-BT), with an outer layer of sodium alginate (ALG) microspheres (AA/CDM-BT-ALG), exhibiting a particle size of 55.1 ± 9.2 µm. In the in vitro dissolution assay, AA/CDM-BT-ALG reduced the cumulative release of AA from 20% to 5.4% in solution (pH = 1.2) for 2 h, demonstrating a good slow-release effect and prolonging the release of the drug in vivo. In addition, AA/CDM-BT-ALG can be released smoothly in the intestinal environment and would not dissolve in gastric juice, which is more favorable to the in vivo absorption of the drug. This might be related to the pH properties of ALG, as it remains stable under acidic conditions but dissolves in neutral to alkaline environments, thereby protecting the drug from being damaged by stomach acid and allowing it to be released smoothly in the intestinal tract. Meanwhile, mechanistic studies revealed that AA/CDM-BT-ALG exerts anti-inflammatory effects by inhibiting the TLR4/MyD88/NF-κB pathway and down-regulating the expression of inflammatory cytokines in colonic tissues and serum, which has a great potential for oral treatment targeting ulcerative colitis. However, this experiment also has certain limitations, such as the lack of discussion on the safety and stability of the carrier material, insufficient long-term safety data, and the complexity of the preparation process, which may make large-scale production difficult to achieve. Raval et al. [8] have prepared a glutathione-coupled asiatic acid-loaded BSA nanoparticles with AA as a novel ligand, and this formulation also showed good slow release, which was able to prolong the in vivo release of AA from 5 h to 24 h, and the AA intracerebral the bioavailability of AA was also increased from 1.26% to 13.4%, which significantly improved the absorption efficiency of AA in vivo. This might be due to the fact that after being modified by glutathione, the nanoparticles are recognized as “endogenous substances”, reducing the efflux mediated by P-gp, and the glutathione coupling enables the nanoparticles to pass through the blood–brain barrier more easily, thereby increasing the brain bioavailability of the drug. AA nano preparations have also shown promising therapeutic effects on cancer, and to improve the efficacy of AA on breast cancer, Dutta et al. [134] prepared AA-loaded poly (lactic acid)-hydroxy acetic acid-based polymer nanoparticles (AA-PLGA NPs) by the multiple emulsion evaporation technique. AA-PLGA NPs were found to be more cytotoxic than AA (IC_50_ of 12.6 μg·mL^−1^, 17.7 μg·mL^−1^, respectively) than AA (IC_50_ of 42.7 μg·mL^−1^, 49.4 μg·mL^−1^, respectively) for both MCF-7 and MDA-MB-231 breast cancer cells Moreover, in vivo experiments showed that tumors in mice treated with AA-PLGA NPs were significantly reduced in vivo compared with those in the AA-treated group (tumor volume shrank from approximately 2 cm^3^ to less than 1 cm^3^), which may result from drug accumulation in the tumor tissue. Other researchers have proposed using AA-PLGA NPs for the treatment of glioblastoma and found that modifying AA-PLGA NPs with transferrin (Tf) can prolong the release time of PLGA NPs (82% release of AA-PLGA NPs within 72 h, Tf-AA-PLGA NPs release 40%), while also enhancing antitumor activity (IC_50_ values for AA-PLGA NPs and Tf-AA-PLGA NPs against U87 cells were 66 ± 2 µM, 52 ± 7 µM), and reduce the toxicity of AA to normal cells (IC_50_: AA-PLGA NPs = 83 ± 13 µM; Tf-AA-PLGA NPs = 199 ± 1 µM) [12]. However, this study still has certain limitations, such as a relatively low Tf modification rate (only 49%), which may affect the drug’s targeting ability and ultimately its therapeutic efficacy. Additionally, the assessment of safety is somewhat limited, as it only uses healthy astrocytes (NHA) as a control group and lacks other types of brain cells, failing to demonstrate broad-spectrum safety. Finally, the preparation method for this formulation is relatively cumbersome, which may make it difficult to achieve large-scale industrial production.

The above studies showed that delivering AA with nanoparticles as a carrier can effectively prolong its release time and retention time in vivo, overcome its shortcomings such as fast elimination and short half-life in vivo, and significantly improve the bioavailability of AA and enhance its pharmacological effects, to better exert its therapeutic efficacy.

### 5.2. Solid Lipid Nanoparticles (SLN)

SLNs are a new type of carrier system used instead of traditional colloidal carriers, in which drugs are encapsulated in a layer of solid lipids stabilized by surface active molecules, often designed to carry poorly water-soluble drugs to improve their bioavailability, with low toxicity, high biocompatibility, high stability and high encapsulation rate [135,136,137].

Islamie et al. [138] showed that AA-SLN formulations administered via naso-cerebral administration were able to exert their neuroprotective effects by inhibiting the hyperphosphorylation of tau, microglia activation, release of pro-inflammatory factors, and oxidative stress, ameliorating Aβ1-42-induced memory deficits in model animals. It is worth noting that the efficacy of intranasal administration of AA-SLN (2.04 mg·kg^−1^) is equivalent to that of oral administration of 302.04 mg·kg^−1^ of AA, indicating that this formulation reduces the drug dosage and can reduce the risk of systemic toxicity to a certain extent. Compared with oral administration of the same dose of AA, intranasal administration of AA-SLN avoids the first-pass effect and, due to its targeted nature, exhibits better neuroprotective effects than AA. AA-SLNs enhance drug bioavailability because the solid lipid matrix of SLNs (composed of biocompatible lipids such as glyceryl monostearate) increases mucosal adhesion and promotes transcellular uptake by nasal epithelial cells. Additionally, encapsulating AA within the lipid core protects it from enzymatic degradation and maintains release kinetics, thereby effectively increasing the drug’s bioavailability in the brain. Another researchers used solvent evaporation and thermal homogenization techniques to prepare three types of AA-loaded solid lipid nanoparticles: AA-loaded glyceryl monostearate SLNs (AA-MS-SLNs), AA-loaded glyceryl distearate SLNs (AA-DS-SLNs), and AA-loaded glyceryl tristearate SLNs (AA-TS-SLNs), and investigated their therapeutic effects on glioma [139]. It was found that the IC_50_ of all three AA-SLNs on U87 MG human glioblastoma cells (38.8–61.0 µM) was lower than that of normal SVG P12 human fetal glial cells (IC_50_ = 59.3–71.6 µM). In addition, the AA-TS-SLNs had the slowest rate of AA release, which may be attributed to the higher hydrophobicity of TS and the retention of hydrophobic drug AA for an effect produced by the long retention time of the hydrophobic drug AA. The results showed that all three AA-SLNs were able to penetrate capillaries and accumulate in tumors and tumor vasculature, and that the formulation was selective and slow-releasing, and was able to significantly promote glioma cell apoptosis without affecting normal cells.

In summary, SLN formulations can prolong the in vivo release time of lipophilic drug AA and endow it with certain targeting properties, thus effectively exerting its effects such as neuroprotection and treatment of glioma.

### 5.3. Liposomes

Dubey [9] prepared chitosan-coated liposomes of AA (CLAA) for the initial phase of oral treatment of AD. In vitro release assay showed that the cumulative release rate of AA from CLAA was 85.3 ± 0.3% within 24 h, which was significantly better than that of pure AA (65.3%), this is because the electrostatic interactions within the lipid bilayer can modulate drug release behavior, thereby enabling more sustained and complete drug delivery, and the in vitro permeation assay found that the adhesive effect of chitosan prolonged the retention time of the drug in the intestinal tract, resulting in a permeation rate of 97.9% ± 4.3% of CLAA within 8 h, which was also significantly higher than that of pure AA (30.90% ± 0.9%). And oral administration of CLAA resulted in a peak serum AA concentration of 9.23 ± 0.34 µg·mL^−1^, which was significantly elevated over AA (3.43 ± 0.12 µg·mL^−1^). The pharmacokinetic parameters showed that oral CLAA (C_max_ = 9.23 ± 0.34 µg·mL^−1^, T_max_ = 6.0 h, AUC_0–t_ = 116.61 ± 4.69 µg·mL^−1^·h) bioavailability was increased by 2.7-fold and 2.9-fold, respectively, compared to AA (C_max_ = 3.43 ± 0.12 µg·mL^−1^, T_max_ = 4.0 h, AUC_0–t_ = 40.51 ± 1.16 µg·mL^−1^·h), and the half-life was extended to 3.49 h. The results indicate that chitosan-coated liposomes enhance drug absorption by protecting the drug from enzymatic degradation. In summary, CLAA can convert AA into formulations with higher bioavailability and neuroprotective potential. Future studies should investigate its effects on molecular pathways in Alzheimer’s disease to enhance its clinical relevance.

### 5.4. Nanostructured Lipid Carriers (NLCs)

NLCs have attracted widespread attention as the second generation of lipid nanoparticles developed from SLNs. NLCs can overcome the deficiencies of lipophilic drugs and improving the bioavailability of insoluble drugs by increasing drug solubility and permeability, decreasing metabolism as well as inhibiting the exocytosis of p-glycoprotein [140].

Halder [141] prepared AA-loaded nanostructured lipid carriers (AA-NLC, particle size 44.1 ± 12.4 nm) by hot melt emulsification using soy lecithin as a surfactant, the in vitro release assay showed that 85.57% of AA in AA-NLC was able to sustain the release within 24 h, demonstrating a good slow-release ability. After intraperitoneal injection of AA-NLC (75 mg kg^−1^), the C_max_ and AUC_0–t_ of AA in rat plasma were found to be elevated by 1.92-fold and 1.28-fold, respectively, and that of AA in brain were found to be 26.63 ± 4.73 µg·g^−1^ and 207.15 ± 32.72 µg·g^−1^·h, which were elevated compared with AA suspension by 2.28-fold and 2.99-fold, and the half-life T½ increased from 6.4 ± 1.8 h to 15.57 ± 9.4 h, which prolonged the retention time of the drug in the body and significantly enhanced the bioavailability of AA. Subsequently, the researchers found that AA-NLC treatment significantly shortened the avoidance latency of AD model rats (from 22.48 s to 7.32 s in the AA suspension-treated group) through the Morris water maze assay, confirming that AA-NLC improves AA bioavailability and enhances its therapeutic efficacy in AD. Another researcher [11] prepared PEG-ylated nanostructured lipid carriers (P-AA-NLC) by solvent diffusion method, aiming to improve intestinal absorption and targeted delivery of AA. In vitro release experiments showed that P-AA-NLC had significant slow-release properties at pH = 7.4, while its release rate in gastric juice was only 0.56%, indicating that it could effectively protect the drug from degradation by gastric acid. In vivo pharmacokinetic studies demonstrated that the relative bioavailability of P-AA-NLC (C_max_ = 67.82 ± 5.05 μg·h^−1^, AUC_0–24h_ = 390.58 ± 56.60 µg) was 1.5-fold higher than that of AA-NLC (C_max_ = 38.54 ± 8.64 μg·h^−1^, AUC_0–24h_ = 263.20 ± 66.55 µg) 1.5-fold increase and significantly enhanced hepatic targeting (fluorescence intensity 1.3-fold of the unmodified vector). P-AA-NLC (32 mg·kg^−1^) significantly reduced serum ALT, AST, and liver tissue MDA levels in CCl4-induced hepatic fibrosis rats, thereby ameliorating liver fibrosis. Zhang [142] prepared an ursodeoxycholic acid (UA)-modified AA nanostructured lipid carrier (UP-AA-NLC) based on UA modification, which endowed AA with certain targeting properties. In vivo near-infrared fluorescence imaging in rats showed that the UP-AA-NLC was still fluorescent at 48 h. Moreover, the hepatic drug concentration in the UP-AA-NLC group was 1.9-fold increased compared with that of the control group, which indicated a prolonged retention time of the drug in vivo and enhanced hepatic targeting.

In summary, AA-NLC formulations can improve AA solubility, prolong in vivo retention time, increase bioavailability, and enhance its targeting, thus enhancing the therapeutic effects of AA on diseases.

### 5.5. Exosomes

The antitumor effects of AA have been extensively documented. The utilization of novel materials, such as nanoparticles and liposomes, to deliver AA and enhance its antitumor activity has also been supported by experimental data. However, the application of these synthetic materials may still pose certain therapeutic risks and toxic effects [143]. Exosomes, a natural nanoscale drug delivery system characterized by intrinsic substance transport capabilities, excellent biocompatibility, and high tumor targeting efficiency, exhibit significant potential as drug carriers, thereby offering novel strategies for cancer therapy [144]. Wu [145] employed differential centrifugation to isolate highly pure exosomes from the supernatants of esophageal cancer cells (KYSE-150 and TE-1). These exosomes were subsequently utilized to load AA, thereby addressing the limitation wherein only a small fraction of AA reaches the lesion post-administration and enhancing the anti-tumor activity of AA. In vitro cytotoxicity assays demonstrated that the cytotoxicity of drug-loaded exosomes (AA-loaded EXOs) was significantly greater than that of free AA against KYSE-150 and TE-1 cells. The LC_50_ values for AA-loaded EXOs were 9.12 ± 2.21 μg·mL^−1^ and 11.34 ± 3.02 μg·mL^−1^ for KYSE-150 and TE-1 cells, respectively, while those for free AA were 17.53 ± 1.32 μg·mL^−1^ and 19.27 ± 1.77 μg·mL^−1^, respectively. This potentiating effect may be attributed to AA being encapsulated within the exosome’s lipid bilayer, which enhances its solubility. Furthermore, exosomes enter cells via endocytosis and possess surface proteins that facilitate efficient cellular uptake. This reduces drug loss, thereby improving bioavailability and ultimately enhancing its cytotoxicity. Experiments on the effect on tumor cell migration rate revealed that AA-loaded EXOs increased the migration inhibition rate of KYSE-150 cells and TE-1 cells by 2.5-fold and 2.9-fold, respectively, compared with AA. The experimental results showed that AA-loaded exosomes had strong cytotoxicity as well as more significant inhibition of cancer cell migration than AA. Recently, substantial advancements have been achieved in tumor-targeting strategies utilizing exosome-based drug delivery systems. However, research on AA encapsulated within exosomes has remained relatively limited in recent years. Additionally, there is a paucity of in vivo experiments to adequately demonstrate the safety of exosome-mediated drug delivery, leading to insufficient data to support its clinical translation. Future investigations should focus on strengthening exosome-related research to provide robust theoretical underpinnings for the clinical application of AA.

### 5.6. Gel Formulations

Gels are semi-solid preparations consisting of a drug and one or more gelling agents (also known as gelling agents or thickeners). Among them, hydrogels are crosslinked polymer chains with 3D network structure composed of hydrophilic polymers, which are characterized by high water content, soft structure, high biocompatibility, degradability, and porousness [146,147], making them an ideal vehicle for dermal drug delivery. Gel formulations are more suitable for topical delivery to the skin compared to the slow release properties of nanoparticles. Incorporation of healing active ingredients into hydrogels can accelerate wound healing and shorten the healing time [10]. Li et al. [148] showed that 3.5% hyaluronic acid (HA) as a hydrogel matrix significantly improved the skin permeability of AA and released up to 79.38 ± 1.27 µg·cm^−2^ in Modified Franz Diffusion Cell, breaking through the limitation of poor penetration through the upper keratinized skin layer. Similarly, the use of L-menthol as an enhancer can also enhance the skin penetration effect of AA (the residual drug content in the skin layer reached 58.75 ± 8.13 μg·g^−1^, nearly double that of the AA control group), thereby improving its efficacy in skin diseases. The carboxyl groups on HA provide a weakly acidic environment conducive to the molecular form of AA, promoting its diffusion. Additionally, HA exhibits adhesive properties that facilitate drug retention and penetration on the skin surface, potentially explaining why HA-based hydrogels enhance AA permeability. Notably, the safety of chemical permeation enhancers such as L-menthol requires further validation to avoid adverse effects that could compromise topical treatment efficacy. Future studies should further validate in vivo pharmacodynamics and toxicology to lay the foundation for clinical translation. Cao’s team [149] prepared novel hydrogel formulations by combining natural melanin nanoparticles (MNPs) and AA, and found that hydrogel formulations containing AA promoted healing and remodeling of skin wounds (On the ninth day, the wound healing rate reached 100% in the AA-gel group, compared to 85% in the control group (without AA)). However, the study did not set up a control group using AA alone, and the absence of an AA-only group made it impossible to distinguish whether the change was caused by AA alone or by AA in combination with MNPs and hydrogels. In addition, researchers [82] prepared a novel cationic chitosan-based polymer hydrogel (AA-gel) loaded with AA, which also has the effect of promoting the healing and remodeling of skin wounds, and in zebrafish wound healing experiments, AA-gel intervention significantly improved the damaged areas of zebrafish, with a healing rate as high as 95%, a 34% improvement over the pure hydrogel group (healing rate of 61%), and it was found through mechanistic studies that AA-gel also reduced inflammatory responses by down-regulating the expression of IL-1β, TNF-α and TGF-β to reduce the inflammatory response. AA-gel has also been shown to have certain antibacterial properties. At a concentration of 20 µg·mL^−1^, AA can reduce the biomass of MRSA bacterial biofilms by 85.66% and inhibit the growth of MRSA bacteria by up to 89.66%. However, AA-gel can increase the antibacterial rate of AA against MRSA to 93%. Zhang et al. [150] have explored for the first time the formation of AA-Mg self-assembled hydrogels coordinated with Mg^2+^. It has been shown that this hydrogel also exhibits excellent antibacterial activity and wound-healing properties. After treatment with the hydrogel, the survival rate of *Escherichia coli* was only 0.46 ± 0.26% (compared to 7.78 ± 1.79% in the control group, indicating a nearly 17-fold enhancement in antibacterial efficacy), the survival rate of *Staphylococcus aureus* was 3.64 ± 0.32% mm (compared to 15.88 ± 2.01% in the control group, representing a 4.4-fold increase in antibacterial efficacy), demonstrating significant antibacterial activity. In wound healing experiments on male Balb/c mice, it was found that after 14 days of AA-Mg hydrogel intervention, the unhealed wound area was only 1.66 ± 1.55%, significantly lower than that in the AA-treated group (62.96 ± 4.54%), which was due to the effect of the synergistic promotion of collagen deposition, granulation tissue and neovascularization by AA and Mg^2+^.

Transfersomes are an advanced drug delivery system based on liposomes modified for the treatment of psoriasis, eczema, and other skin diseases. On this basis, Abdul et al. [151] developed an AA-loaded Transliposome gel (AA-TL) for dermal administration by combining the transferosome with a gel. It was found that the conventional AA gel formulation penetrated the rat skin by 32.48 ± 2.42%, whereas the optimized AA-TL was able to increase the AA skin penetration to 79.83 ± 4.52%. Dermatokinetic analysis revealed that AA-TL (T_skin max_ = 162.44 ± 6.42 µg·cm^−2^, AUC_0–8_ = 708.18 ± 8.85 µg·cm^−2^·h) was significantly more effective than the conventional AA gel formulation (T_skin max_ = 84.55 ± 3.78 µg·cm^−2^, AUC_0–8_ = 62.64 ± 7.32 µg·cm^−2^·h) in dermal application. The flexible structure of transfersomes and the edge-activated agents they contain facilitate carrier penetration through the stratum corneum, thereby enhancing drug transdermal permeability. Additionally, pharmacokinetic parameters demonstrate the superiority of AA-TL, likely due to transfersomes enhancing drug distribution within the skin’s lipid bilayer. Another researcher [152] investigated the in vitro permeability of AA in the form of a gel in a transferosome by using a Franz diffusion cell. A novel transferosome gel (TW80AATG) was made by mixing soy lecithin with Tween 80 in a 50:50 ratio and 0.3% *w*/*w* AA by the high-pressure homogenization technique. The study used Strat-M^®^ film as a hypothetical skin and found that at 8 h of administration, the AA permeation in TW80AATG and the flux through the Strat-M^®^ film were 8.53 ± 1.42% and 0.024 ± 0.008 mg·cm^−2^·h^−1^, respectively, which were significantly higher than that of the non-transfersomal gel (0.73 ± 0.44~3.13 ± 0.46%, 0.002 ± 0.001~0.010 ± 0.002 mg·cm^−2^·h^−1^). Mahadev [153] developed a chitosan-coated virgin coconut oil-asiatic acid-loaded nanoemulsion gel (CS-ASA-NEG) to alleviate the difficulties in wound healing caused by delayed healing as well as complications such as microbial infections, in combination with the potent healing activity of AA. Skin permeation experiments revealed that the maximum flux of CS-ASA-NEG was 159.10 ± 0.08 µg·cm^−2^·h^−1^, which was a significantly higher performance than the commercially available gel (154.60 ± 0.21 µg·cm^−2^·h^−1^). At 20 days of use of the preparation, the wound healing rate in mice was 99.86% and almost healed. The success of this formulation stems from the effective utilization of nanoemulsion technology to enhance the solubility and permeability of hydrophobic drugs, coupled with the synergistic effects of adhesion, permeation promotion, and activity enhancement provided by the chitosan coating. Compared to traditional carrier gels, nanoemulsion gels may offer advantages in drug loading capacity and suitability for superficial wound applications. The excellent healing ability of CS-ASA-NEG suggested that synergizing AA with virgin coconut oil and chitosan could enhance its healing activity, but its efficacy still needs to be further verified by clinical trials.

It can be seen that gel preparations can significantly enhance the skin permeability of AA, which can significantly help to improve the efficacy of skin diseases. At the same time, some gel preparations can also enhance the antimicrobial activity of AA, reducing the risk of bacterial infection in wounds and indirectly helping wound healing.

The novel drug delivery system exhibits promising application prospects by substantially enhancing the bioavailability of AA, imparting drug targeting capabilities, extending the duration of drug action, achieving sustained and controlled release, and improving the retention time of the drug within the body. This fundamentally overcomes the limitations imposed by the physicochemical properties of AA, thereby enhancing its therapeutic efficacy. However, most of the current novel drug delivery systems rely on conventional technologies, and there is a gap in research on emerging specific carriers such as exosomes [154], DNA nanostructures [155], and bioreactive hydrogels [156]. Currently, most research on novel formulations remains confined to mouse model experiments, lacking preclinical pharmacokinetic, toxicological studies, and clinical trial data, which limits their clinical translation. Additionally, the preparation methods for certain novel formulations are complex and difficult to scale up (e.g., AA/CDM-BT-ALG, AA-Mg self-assembled hydrogels), which impedes the transition from laboratory research to large-scale industrial production. Future efforts should focus on safety studies of new formulations and the exploration of simpler and more efficient preparation methods. In addition, it is possible to further optimize the stability and degradation rate of the formulation to adapt to different disease models. Additionally, it is necessary to conduct in-depth research into the specific mechanisms by which different delivery carriers enhance the bioavailability of AA, and explore novel carrier materials with higher biocompatibility (such as exosomes, bioactive hydrogels, etc.) to provide a more robust scientific basis for the broader and more precise application of novel formulations. At the same time, we will draw on the experience of successfully marketed natural product nanomedicines (e.g., paclitaxel albumin-binding nanoparticles Abraxane^®^) to promote the clinical translation of novel AA formulations.

**Figure 6:**
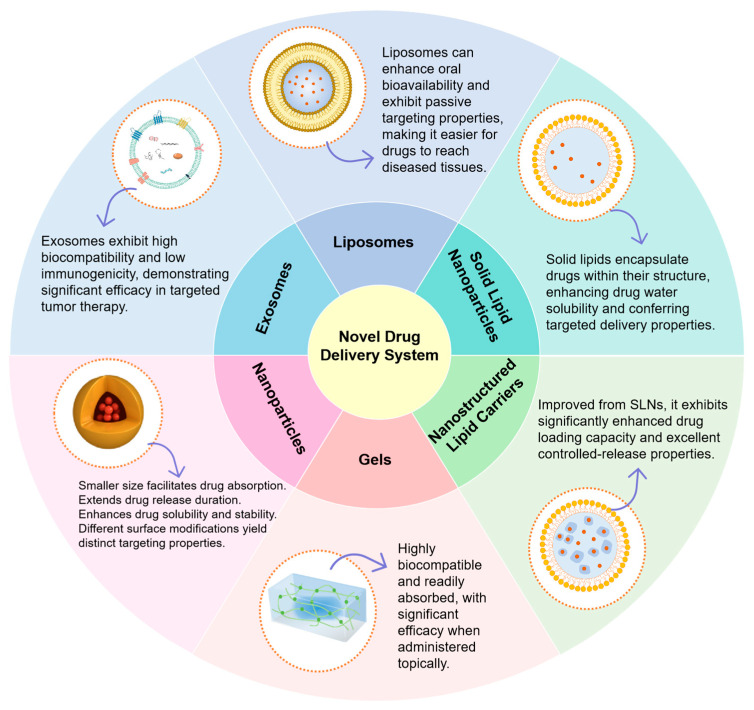
Schematic diagram of the new delivery system.

In order to systematically summarize the recent progress in AA formulation research, this paper reviews various types of new formulations, such as nanoparticles, solid lipid nanoparticles, liposomes, nanostructured lipid carriers, exosomes, and gels (Figure 6). These formulations have shown great potential in improving the solubility and bioavailability of AA. Table 4 provides a detailed summary of the preparation methods, carrier materials, release models, and efficacy of each formulation, providing a reference for the future development and optimization of new AA formulations.

## 6. Clinical Transformation and Challenges

The clinical translation of AA has long been limited by its physicochemical properties and complex pharmacological profile. In recent years, with the research progress of Chinese medicine theory, the pharmacological effects of AA and its mechanisms are becoming increasingly clear, and the emergence of AA derivatives and new dosage forms has shown good results in improving AA bioavailability, overcoming the limitations of poor water solubility and low bioavailability of AA, and providing solid data support for its wide application in clinical practice. However, the clinical translation of AA continues to encounter certain challenges, such as toxicity concerns, scalability limitations, and variable pharmacokinetics. The low water solubility of AA leads to its low oral bioavailability, which requires structural modification or a novel delivery method to improve the efficacy, but the current toxicological evaluation of AA and its derivatives is not sufficient, and the safety of the new dosage form using polymer materials is still to be considered, which hinders progression to the clinical trial stage, so there is no clinical study to report the safety and toxicity of AA yet. Secondly, AA’s current novel formulations such as nanoparticles, solid lipid nanoparticles, and nanoliposomes are complex to prepare and have disadvantages including difficulty in scale-up, biocompatibility concerns, low drug loading, storage instability [157], and high production costs, leading to their poor scalability. Although researchers have developed many well-designed drug nanocarriers, only a few such formulations have been successfully translated into the clinic [158], and in addition the size of the nanoparticles affects the pharmacokinetic parameters of the drug, with blood half-life, targeting, and cellular uptake of the treatment being affected by the nanoparticle size [130], and thus the new dosage forms of AA are still limited to the laboratory stage. In order to break through the barriers to clinical translation of AA as soon as possible, the successful experience of paclitaxel albumin nanoparticles (Abraxane^®^), which have been produced on a large scale, can be used to develop a low-energy preparation process using a quality by design (QbD) strategy [159], the use of natural proteins from diverse sources for excipients [160], and the standardization of the process. The sophisticated regulatory frameworks of the U.S. FDA and the European Union’s EMA offer comprehensive guidance for the transformation of such natural products. By integrating the Quality by Design (QbD) concept with the requirements outlined in the FDA/EMA guidelines for plant-derived drug development, process-robust AA nano-delivery systems can be developed to overcome existing technological bottlenecks, thereby providing additional references for the clinical translation of AA.

## 7. Conclusions

Asiatic acid, as the active ingredient of the traditional Chinese medicine *C. asiatica*, has a wide range of pharmacological effects, and its multi-target mechanisms underlie these diverse effects, especially in anti-tumor applications. Studying the mechanism of action of asiatic acid can provide valuable insights for its clinical application. The clinical application of asiatic acid has been limited by poor solubility, rapid metabolism, and low bioavailability. However, with the research of derivatives as well as novel dosage forms, the water solubility and bioavailability of asiatic acid have been significantly improved, but there are still some shortcomings, including the structure modification process of asiatic acid being complicated, the nanoformulations prepared from novel materials having low drug loading capacity, which is inadequate for conditions requiring high drug loads, solid lipid-based formulations potentially suffering from drug leakage during the storage process, and the retention time of some hydrogel formulations on the skin surface being short, necessitating frequent administration. Therefore, addressing the challenges of complex derivative preparation, low drug loading, and poor stability of novel dosage forms warrants further attention. Furthermore, current research on asiatic acid derivatives and novel dosage forms remains limited to in vitro and in vivo experimental stages, and their safety and stability in the human body require further validation. In the future, AA derivatives and novel dosage forms require additional evaluation through clinical trials and toxicological studies, Moreover, overcoming the limitations of current novel dosage forms and exploring AA in combination with advanced carrier materials holds significant promise for advancing research.

## Figures and Tables

**Table 1:** Overview of the anti-inflammatory mechanisms of asiatic acid.

Types of Inflammation	Animal/Cell	Dosages	Pathway	Results	Ref.
Endometritis	mouse endometrial epithelial cells	20, 40, 80 µM	TLR4/NF-κB, PPARγ	TNFα ↓, IL1β ↓, PGE2 ↓, NO ↓	[17]
Neuroinflammation	SH-SY5Y cells	10 nM	NLRP3	IL-1β ↓, TNFα ↓, Caspase-1 ↓, mtROS ↓	[18]
Rheumatoid arthritis	RA-FLS cells	80, 100 µM	Nrf2/HO-1, NF-κB	NF-κB ↓, Bax ↑, caspase-3 ↑, Bcl-2 ↓, Proliferation of RA-FLS ↓	[19]
Osteoarthritis	Chondrocytes cells	5, 10, 25 µM	PPARγ, NF-κB	iNOS ↓, Cox2 ↓, Mmp13 ↓	[20]
Alcoholic fatty hepatitis	Raw264.7 cells C57BL/6J mice	5, 25, 50 mg·kg^−1^	NF-κB	NF-κB-Pp65 ↓, TNF-α ↓, IL-1β ↓, IL-6 ↓	[21]
Atopic dermatitis	HaCaT cells BALB/c mice	5, 10, 20 μg·mL^−1^ 30, 75 mg·kg^−1^	NF-κB, MAPK	COX-2 ↓, CXCL9 ↓, IL-6 ↓, TNF-α ↓, IL-8 ↓, NF-κB ↓, MAPK ↓, p-p38 ↓, p-JNK ↓, p-ERK1/2 ↓	[22]
Inflammations	BV-2 cells	12.5 µM	NF-κB, NLRP3	IL-1β ↓, IL-6 ↓, IL-18 ↓, lncRNA ↑, TVX1 ↑, p-p65 ↓ Caspase 1 ↓	[23]
	BV-2 cells	1, 10, 100 µM	Sirt1/NF-κB	Sirt1 ↑, NF-κB-p65 ↓, TNF-α ↓, IL-1β ↓, IL-6 ↓, NO ↓, iNOS ↓	[24]
	ICR mice	1, 5, 10 mg·kg^−1^	NF-κB	CAT ↑, SOD ↑, GPx ↑ MDA ↓, iNOS ↓, COX-2 ↓, NF-κB ↓	[25]
Ulcerative colitis	THP-1 Cells female C57BL/6 mice	15, 30, 60 µM 3, 10, 30 mg·kg^−1^	—	TNF-α ↓, IL-1β ↓, IL-6 ↓, IFN-γ ↓, NLRP3 ↓	[26]
*Salmonella*-induced colitis	Balb/c mice	10 mg·kg^−1^	—	claudin-2 ↑, claudin-7 ↑ IL-1β ↓, IL-6 ↓, TNF-α mRNA ↓	[16]
Ulcerative colitis	Wistar rats	20, 40 mg·kg^−1^	NF-κB	TNF-α ↓, IL-1β ↓, PGE2 ↓, MCP-1 ↓, NF-κB p65 ↓	[27]
Acute pulpitis	Wistar rats	0.5%, 1%, 2.0% (*v*/*w*)	Nrf2/ARE, NF-κB/MAPK	NF-κB ↓, MAPK ↓, TNF-α ↓, MDA ↓, CGRP ↑, SOD ↑, β-endorphin ↑	[28]
Cystitis	Wistar rats	30 mg·kg^−^^1^·day^−^^1^	NF-κB, NLRP3	IL-1β ↑, IL-6 ↑, NGF ↑, TNF-α ↑, ORM1 ↑, HPX ↑, MDA ↓	[29]

Note: ↑ indicates an upward adjustment or increase; ↓ indicates a downward adjustment or decrease.

**Table 2:** Overview of the anti-tumor mechanisms of asiatic acid.

Types of Cancer	Animal/Cell	Dosages	Pathway	Results	Ref.
Breast cancer	HUVE Cells BALB/c mice	40 µM 50 mg·kg^−1^	VEGF/VEGFR2	VEGF ↓, VEGFR2 ↓, ERK1/2 ↓, p-Src ↓, p-FAK ↓	[36]
	MCF-7, MDA-MB-231 Cells	—	PI3K/AKT	WAVE3 ↓, P53 ↓, p-PI3K ↓, p-AKT ↓	[37]
	MCF-7 Cells	40, 80, 160 µM	AMPK	AMPKα ↓, ROS ↑, ATP ↓, P-gp ↑	[41]
Nasopharyngeal carcinoma	NPC-bm, NPC-039 Cells	50, 75 µM	p38/MAPK	Bax ↑, p-38 ↑, JNK ↑, Caspase-3 ↑	[42]
	TW-01, SUNE5-8F Cells	—	—	STAT3 ↓, Claudin-1 ↓, caspase-3 phosphorylation ↑	[49]
Non-small cell lung cancer	A549, DDP Cells	—	MALAT1/miR-1297/p300/β-catenin ↓,	MALAT1 ↓, p300 ↓, β-catenin ↓, MDR1 ↓, cleaved caspase-3 ↑, miR-1297 ↑,	[50]
	A549, NSCLC Cells	50, 100 µM	—	miR-1290 ↑BCL2 ↓	[51]
	A549, H460, HNSCLC Cells	30, 60 µM	PI3K/Akt/mTOR, MAPK/ERK	MAPK/ERK ↓, Akt ↓, VEGF ↓, COX-2 ↓, PI3K ↓, mTOR ↓, HIF-1 ↓	[40]
Lung cancer	A549 cells, H1299 cells C57BL/6J mice	20, 40, 80 µM 50, 100 mg·kg^−1^	—	Mitochondrial functions ↓, PARP ↑, caspase-9 ↑, caspase-3 ↑	[52]
	A549 Cells	40 µmol·L^−1^	TGF-β1/Snail, Wnt/β-catenin	E-cadherin ↑, Snail ↓, N-cadherin ↓, vimentin ↓, β-catenin ↓, p-GSK-3β ↓,	[53]
Ovarian cancer	SKOV3, OVCAR-3 cells	10, 40 μg·mL^−1^	PI3K/Akt/mTOR	PI3K ↓, Akt ↓, mTOR ↓	[38]
Renal cell carcinoma	786-O, A-498, Caki-1, ACHN Cells	40 µM	ERK/p38MAPK	p-ERK1/2 ↓, p-p38MAPK ↓, MMP-15 ↓	[39]
Tongue cancer	BALB/cANNCjr nu/nu mice	40 µM 15 mg·kg·d^−1^	Grp78/IRE1α/JNK	Grp78 ↑, P-JNK ↑, P-IRE1α ↑, caspase-3 ↑, Bcl-2 ↓	[54]
Prostate cancer	22Rv1, PC3, DU145 Cells	20, 30 µM	MZF-1/Elk-1/Snail	MZF-1 ↓, Elk-1 ↓, Snail ↓, MEK3/6-p38/MAPK ↓	[55]
Skin cancer	SK-MEL-2 Cells	20 µM	—	ROS ↑, bax ↑, caspase-3 ↑	[56]

Note: ↑ indicates an upward adjustment or increase; ↓ indicates a downward adjustment or decrease.

**Table 3:** Summary of the changes in efficacy and physical properties of asiatic acid after structural modification.

Modification Site	Modification Type	Physical Property Changes	Pharmacological Effect Changes	Ref.
C-2, C-3, C-23; C-28	Acetylation; Esterification	Stability ↑ (No decomposition at 37 °C and −20 °C)	Anti-tumor activity ↑ (Decreased IC_50_ for multiple tumor cells); Inhibition of angiogenesis ↑; Toxicity to normal cells ↓; Ability to induce cancer cell apoptosis ↑	[99,102,103]
C-2, C-3, C-23; C-28	Acetylation; Rhodamine B conjugation	Liposolubility ↑	Anti-tumor activity ↑	[104]
C-23	Esterification	Liposolubility ↑	Anti-tumor activity ↑ (The smaller the amide group at the C-23 position, the stronger the antitumor activity of the derivative.)	[3]
C-11; C-28	Aniline substituent; Amidation	Water solubility ↑; Membrane permeability ↑	Anti-tumor activity ↑ (After C-28 amidation, the IC_50_ of the compound against HepG2 liver cancer cells decreased from 34.9 µM to 5.97 µM.)	[5]
C-28	Amidation	—	Anti-tumor activity ↑ (Increased toxicity to HepG2 and SGC7901 cells); Inhibition of VEGF secretion and VEGFR phosphorylation	[109,110]
C-2, C-3, C-23; C-12; C-28	Acetylation; Fluorination; Amidation	—	Anti-proliferative activity ↑	[112]
C-28	Esterification; Amidation	Liposolubility ↑; Water solubility ↑	Activity of the derivatives against Rabbit Muscle GPa ↑ (When a lipophilic derivative is introduced at the C-28 position); Activity of the derivatives against Rabbit Muscle GPa ↓ (When amino acid derivatives are introduced at the C-28 site)	[116]
C-2, C-3, C-23; C-28	Sulfonylation; Amidation	Water solubility ↑	Inhibitory activity against hCA VA ↑	[117]
C-2; C-28	Oxidation; Anhydride formation	—	Cognitive enhancement ability ↑	[118]
C-2, C-3, C-23; C-28	Acetylation; Amidation	Water solubility ↑	Induce osteogenic differentiation of hPDLSCs cells ↑ (Osteogenic activity when dimethylaminopropylamine was introduced at C-28 site ↑; Osteogenic activity when introducing long-chain alkyls ↓)	[120]
C-28	Esterification	Water solubility ↑	Cytotoxicity ↓; Induction of osteogenic potential ↑ (Osteogenesis can be induced at low concentrations (1–10 µM))	[121]

Note: ↑ indicates increase or enhancement; ↓ indicates decrease or reduction.

**Table 4:** Comparison of preparation techniques and performance of asiatic acid formulations.

Types of Formulations	Formulation Name	Carrier Material/Structure	Preparation Technology	Release Model	Particle Size	Drug Loading/Content	Encapsulation Efficiency	Route of Administration	**Effect**	**Ref.**
Nanoparticle	AA/CDM-BT-ALG	CDM-BT-ALG	Solvent evaporation technique	Anomalous Diffusion	37.8 ± 7.1 nm	13.0 ± 1.0%	99.3 ± 7.5%	Oral administration	Increase intracellular drug concentration; prolong drug release time in the body	[133]
	AA-loaded BSA NPs	Bovine Serum Albumin, BSA	Modified desolvation technique	Biphasic Release	228.66 ± 2.51 nm	16.33 ± 1.52%	60.00 ± 1.00%	Injection administration	Extended drug release time in the body; 10-fold increase in drug bioavailability in the brain	[8]
	AA-PLGA NPs	PLGA (polylactic acid-hydroxyacetic acid copolymer)	Multiple emulsion solvent evaporation technique	Biphasic Release	359.6 nm	6.08 ± 0.29%	65.63 ± 1.88%	Injection administration	Selective cytotoxicity, reducing the volume and mass of breast tumors in mice; prolonging the release time of drugs in the body.	[134]
	Tf-AA-PLGA NPs	PLGA; Transferrin (Tf)	Single Emulsion-Solvent Evaporation method	Biphasic Release	149 ± 2 nm	3.3 ± 0.1%	66 ± 3%	Injection administration	Tf modification significantly enhances the uptake of nanoparticles in U87 cells, improving antitumor activity and reducing toxicity to healthy cells.	[12]
Solid lipid nanoparticle	AA-SLN	Glyceryl monostearate	Hot Melt emulsification	Anomalous Diffusion	189.27 ± 4.22 nm	2.26 mg·mL^−1^	—	Intranasal administration	Combined with intranasal administration, this avoids the first-pass effect and increases the concentration of the drug in the brain.	[138]
	AA-MS-SLNs; AA-DS-SLNs: AA-TS-SLNs	Glyceryl monostearate; Glyceryl distearate; Glyceryl tristearate	Solvent evaporation and hot homogenisation technique.	Anomalous Diffusion	141.7 ± 1.7 nm; 141.3 ± 2.5 nm; 126.9 ± 0.5 nm	5.0 ± 0.25%; 2.1 ± 0.30%; 3.1 ± 0.12%	98. ± 0.05%; 98.1 ± 0.60%; 99.9 ± 0.04%	Injection administration	Extending drug action time; SLNs enhance AA’s targeted toxicity to glioblastoma cells while reducing damage to normal cells.	[139]
Liposomes	CLAA	Soybean lecithin; Chitosan; Cholesterol	Solvent evaporation technique	Higuchi model	209.8 nm	68 ± 0.04%	71.2 ± 0.1%	Oral administration	Extended drug retention time in the intestine, AUC increased by 2.9 times, T_1_/_2_ extended to 3.49 h; improved drug permeability in the intestine.	[9]
Nanostructured lipid carriers	AA-NLC	Glyceryl Monostearate; Oleic Acid; Soybean Lecithin	Hot-melt emulsification technique	Anomalous Diffusion	44.1 ± 12.4 nm	20%	73.41 ± 2.53%	Injection administration	Enhance the penetration and absorption of AA through the blood–brain barrier; Extending the retention time of the drug in the body, the C _max_ and AUC_0–t_ in the brain were increased by 2.28 and 2.99 times, respectively, compared to the AA suspension, and T_1_/_2_ was extended to 15.57 h.	[141]
	P-AA-NLC	Glyceryl Monostearate; Oleic Acid; PEG2000-SA	Solvent Diffusion method	Anomalous Diffusion	160.50 ± 4.16 nm	19.03 ± 0.18%	93.3 ± 0.9%	Oral administration	Protect drugs from dissolution by stomach acid, improve drug bioavailability, and give drugs a certain degree of liver targeting.	[11]
	UP-AA-NLC	UA-PEG-SA (Ursodeoxycholic acid-polyethylene glycol-stearic acid); Glyceryl Monostearate; Oleic Acid	Solvent Diffusion method	Ritger–Peppas model	159.7 ± 4.9 nm	10.53 ± 0.10%	77.44 ± 0.69%	Oral administration	Increases drug concentration in the liver (6.2 times higher than free AA) and prolongs drug retention time in the body.	[142]
Exosomes	AA-loaded EXOs-K; AA-loaded EXOs-T	EXOs-K;EXOs-T	Differential Ultracentrifugation	Biphasic Release	122.7 ± 2.8 nm; 111.2 ± 3.4 nm	7.9 ± 1.2%; 7.5 ± 0.8%	—	Oral administration	Slow and small release in blood or normal cell environment, continuous release in tumor sites	[145]
Gel formulations	AA hydrogel	Hydrogel containing 3.5% hyaluronic acid	Physical mixing method	Zero-level release pattern	—	2.0%	—	Transdermal administration	Improve the penetration of drugs into the deep layers of the skin	[148]
	Hybrid structural color hydrogel patch	FMA; AG; MNPs	Thermal Melting Infusion	Light/NIR-triggered release	—	1 mg·mL^−1^	—	Topical administration	Combining photothermal effects to control drug release; promoting wound healing and remodeling.	[149]
	AA-gel	Chitosan; Gelatin	Solvent casting method	Higuchi model	—	20 µg·mL^−1^	—	Topical administration	Accelerate wound healing, control drug release, and enhance antibacterial capacity.	[82]
	AA-Mg self-assembled hydrogels	Mg^2+^; Bacterial Cellulose	Self-assembly technology	Anomalous Diffusion	—	7.7 mg·mL^−1^	—	Topical administration	Maintain the sustained release of AA; accelerate wound healing rate; synergize with Mg^2+^ to exert anti-inflammatory and antibacterial effects.	[150]
	AA-TL	Lipoid S100; cholesterol; triethanolamine; Carbopol 934	Thin-Film Hydration -Sonication	Higuchi model	—	—	87.66 ± 2.12% (In the liposomes in the gel)	Topical administration	Improve the penetration efficiency of drugs into the skin; form a drug deposit layer in the skin to prolong the duration of action.	[151]
	TW80AATG	Soybean Lecithin; Tween 80; Span 80; sodium deoxycholate	High-Pressure homogenization method;	Higuchi model	—	2.80 ± 0.05 mg·g^−1^	—	Topical administration	Improve drug penetration and flux into the skin;	[152]
	CS-ASA-NEG	Oil Phase; Aspirin, ASA; Chitosan; Carbopol 934	High-pressure homogenization method; Nano-emulsion gelation	Higuchi model	131.80 ± 0.33 nm (In the liposomes in the gel)	0.3%	131.80 ± 0.33 nm (In the liposomes in the gel)	Topical administration	Improve drug penetration into the skin; increase drug retention time at the site of inflammation; improve biocompatibility.	[153]

## Data Availability

No new data were created or analyzed in this study. Data sharing is not applicable to this article.
